# Prognostic impact of low muscle mass and visceral adiposity in patients with cirrhosis undergoing transjugular intrahepatic portosystemic shunt: a retrospective cohort study

**DOI:** 10.3389/fnut.2026.1817771

**Published:** 2026-07-02

**Authors:** Zhang Wen, Jia Yuan, Yong Li, Ying Liu, Xinyu Wang, Shuyue Tuo, Meijuan Shi, Jinhai Wang, Shejiao Dai, Lu Li, Xinxing Tantai

**Affiliations:** 1 Department of Gastroenterology, The Second Affiliated Hospital of Xi'an Jiaotong University, Xi’an, China; 2 Clinical Research Center for Gastrointestinal Diseases of Shaanxi Province, The Second Affiliated Hospital of Xi'an Jiaotong University, Xi’an, China; 3 State Key Laboratory of Holistic Integrative Management of Gastrointestinal Cancers and National Clinical Research Center for Digestive Diseases, Xijing Hospital of Digestive Diseases, Fourth Military Medical University, Xi'an, Shaanxi, China; 4 Department of Radiology, The Second Affiliated Hospital of Xi'an Jiaotong University, Xi’an, Shaanxi, China

**Keywords:** cirrhosis, low muscle mass, mortality, prognosis, TIPS (transjugular intrahepatic portosystemic shunt), visceral adiposity

## Abstract

**Objective:**

The prognostic value of low muscle mass and visceral adiposity (LMM-VA) in patients with cirrhosis undergoing transjugular intrahepatic portosystemic shunt (TIPS) has not yet been investigated. We aimed to define LMM-VA using computed tomography-based skeletal muscle index (SMI) and visceral-to-subcutaneous adipose tissue ratio (VSR), and to examine its association with post-TIPS outcomes.

**Methods:**

This retrospective cohort study included adult patients with cirrhosis undergoing TIPS. LMM-VA was defined as the coexistence of low muscle mass (SMI < 44.7 cm^2^/m^2^ in males and < 32.5 cm^2^/m^2^ in females) and visceral adiposity (VSR ≥ 1.21 in males and ≥ 0.48 in females). Associations between LMM-VA and post-TIPS outcomes were evaluated using cumulative incidence curves and univariable and multivariable regression models.

**Results:**

A total of 228 patients with cirrhosis were included in the study, 54 (23.7%) of whom had LMM-VA. During a median follow-up of 29.2 months, patients with LMM-VA showed a significantly higher cumulative incidence of both all-cause mortality (*p* < 0.001) and liver-related mortality (*p* < 0.001). LMM-VA was significantly associated with post-TIPS all-cause mortality in both the univariable (hazard ratio [HR]: 3.62, 95% confidence interval [CI]: 1.65–7.91) and multivariable Cox regression analyses (HR: 3.21, 95% CI: 1.32–7.80). Subgroup, sensitivity, and stratified analyses further confirmed the robustness of this association. Additionally, LMM-VA demonstrated an independent prognostic value for liver-related mortality but not for either new-onset or further decompensation. The significant association between LMM-VA and all-cause mortality was lost when LMM-VA was defined by either a visceral adipose tissue area of ≥ 100 cm^2^ or body mass index of ≥ 25.0 kg/m^2^.

**Conclusion:**

LMM-VA is significantly associated with increased post-TIPS mortality in patients with cirrhosis and may serve as a promising prognostic marker that requires further validation.

## 1 Introduction

Liver cirrhosis is the terminal stage of various chronic liver diseases, accounting for approximately 2.4% of global deaths and posing a major global health burden (1). The major etiologies of cirrhosis include viral hepatitis, alcohol-related liver disease, and the increasingly prevalent metabolic dysfunction-associated steatotic liver disease (MASLD). Cirrhosis can be classified into compensated and decompensated stages based on the occurrence of complications such as variceal bleeding, ascites, and hepatic encephalopathy (HE). Among these complications, portal hypertension is the central mechanism driving decompensation and a key determinant of the markedly increased mortality in affected patients (2). Currently, the main therapeutic approaches for reducing portal pressure include the use of non-selective *β*-blockers (NSBBs) and transjugular intrahepatic portosystemic shunt (TIPS) placement (2). The TIPS procedure is a minimally invasive and highly effective intervention for reducing portal pressure. It is widely used in clinical practice for the management of portal hypertension–related complications, particularly variceal bleeding, refractory ascites, and hepatic hydrothorax (2). Compared with standard of care, TIPS has been shown to reduce the incidence of further decompensation events and improve survival in select patients with cirrhosis (3). However, due to the inherent heterogeneity of cirrhosis, not all patients achieve favorable outcomes after TIPS, with the 1-year survival rate being only about 60% (4, 5). Indeed, Li et al. (5) have reported that the predictive performance of 10 existing prognostic models was generally suboptimal, highlighting the need to explore novel prognostic factors to improve risk stratification and clinical management.

Patients with cirrhosis, particularly those in the decompensated stage, frequently experience various alterations in body composition, such as sarcopenia or sarcopenic obesity. Sarcopenia is characterized by reduced muscle mass and function, and has an estimated prevalence of approximately 40% in patients with cirrhosis (6). Several studies have suggested that pre-procedural sarcopenia is significantly associated with an increased risk of complications and mortality following TIPS (4, 7). However, other studies have reported no significant association between sarcopenia and post-TIPS HE or mortality (8, 9). Furthermore, alterations in adipose tissue are a common accompanying condition in patients with cirrhosis and sarcopenia. Approximately 13–20% of patients with cirrhosis present with substantial muscle wasting concomitant with excess visceral fat accumulation—sarcopenic obesity (10, 11), and this phenotype has been associated with a higher risk of mortality than either sarcopenia or obesity alone (10, 11).

To date, there is no unified consensus on the definition of sarcopenic obesity in patients with cirrhosis, primarily due to heterogeneity in diagnostic methods and the use of inconsistent cutoffs for both sarcopenia and obesity (12). While obesity is commonly defined using body mass index (BMI) thresholds, this approach is suboptimal under cirrhotic conditions because BMI is confounded by fluid retention (i.e., ascites and peripheral edema), ultimately failing to reflect differences in fat distribution. Cross-sectional imaging techniques, such as computed tomography (CT), which directly quantify body composition, including skeletal muscle mass, visceral adipose tissue (VAT), and subcutaneous adipose tissue (SAT), can provide objective evaluation of a patient’s nutritional and metabolic disorders (13). Although previous studies have indicated that VAT and SAT may have differing prognostic implications in cirrhosis, the current evidence remains inconsistent (14, 15). The relative distribution of adiposity—expressed as the VAT-to-SAT ratio (VSR)—may serve as a more accurate indicator of visceral obesity than the absolute areas of VAT or SAT alone, particularly as VSR has been shown to reflect histologic VAT inflammation (16). The association between low muscle mass and visceral adiposity (LMM-VA) and clinical outcomes after TIPS in patients with cirrhosis remains unknown. Therefore, this study investigated the association between LMM-VA—defined by CT-quantified skeletal muscle index (SMI) and VSR—and long-term mortality as well as cirrhosis-related decompensation events following TIPS.

## 2 Methods

### 2.1 Study design and patient selection

This retrospective cohort study included adult patients (≥18 years old) with cirrhosis who underwent TIPS at the Second Affiliated Hospital of Xi’an Jiaotong University between January 1, 2018, and January 20, 2025, and who had undergone an abdominal CT scan within 1 month prior to the procedure. The diagnosis of cirrhosis was established based on findings from laboratory tests in combination with those from imaging and/or liver biopsy. The indication for TIPS placement was recurrent variceal bleeding, confirmed by upper endoscopy. Patients were excluded according to the following criteria: (1) Unsuccessful TIPS procedures due to technical difficulties or other reasons; (2) TIPS performed for causes other than cirrhosis, such as Budd–Chiari syndrome or idiopathic portal hypertension; (3) Indication for TIPS being non-variceal bleeding, such as recurrent or refractory ascites; (4) Presence of severe medical comorbidities that could affect prognosis, including hepatocellular carcinoma or other active malignancies, advanced pulmonary disease (i.e., obstructive pulmonary disease classified as stage III–IV according to the Global Initiative for Chronic Obstructive Lung Disease), chronic kidney disease (stage III or higher, or requiring dialysis), or severe cerebrovascular disease; (5) Abdominal CT scans lacking the L3 vertebral level, of inadequate image quality, or performed more than 1 month before TIPS; (6) Incomplete or missing imaging or clinical data; and (7) Patients who were lost to follow-up within 3 months after the procedure. Data on baseline patient characteristics were collected, including demographic and clinical data, serum biochemical parameters, imaging-based measurements, and TIPS-related procedural information. All patients were followed by reviewing medical records or via telephone interviews until liver transplantation, death, or the last available follow-up on June 15, 2025. This study was approved by the institutional review board of our hospital (No. 2022032), and all procedures were conducted in accordance with the ethical principles of the Declaration of Helsinki. Due to the retrospective design of the study, the requirement for written informed consent was waived.

### 2.2 Measurement of body compositions

**Figure 1:**
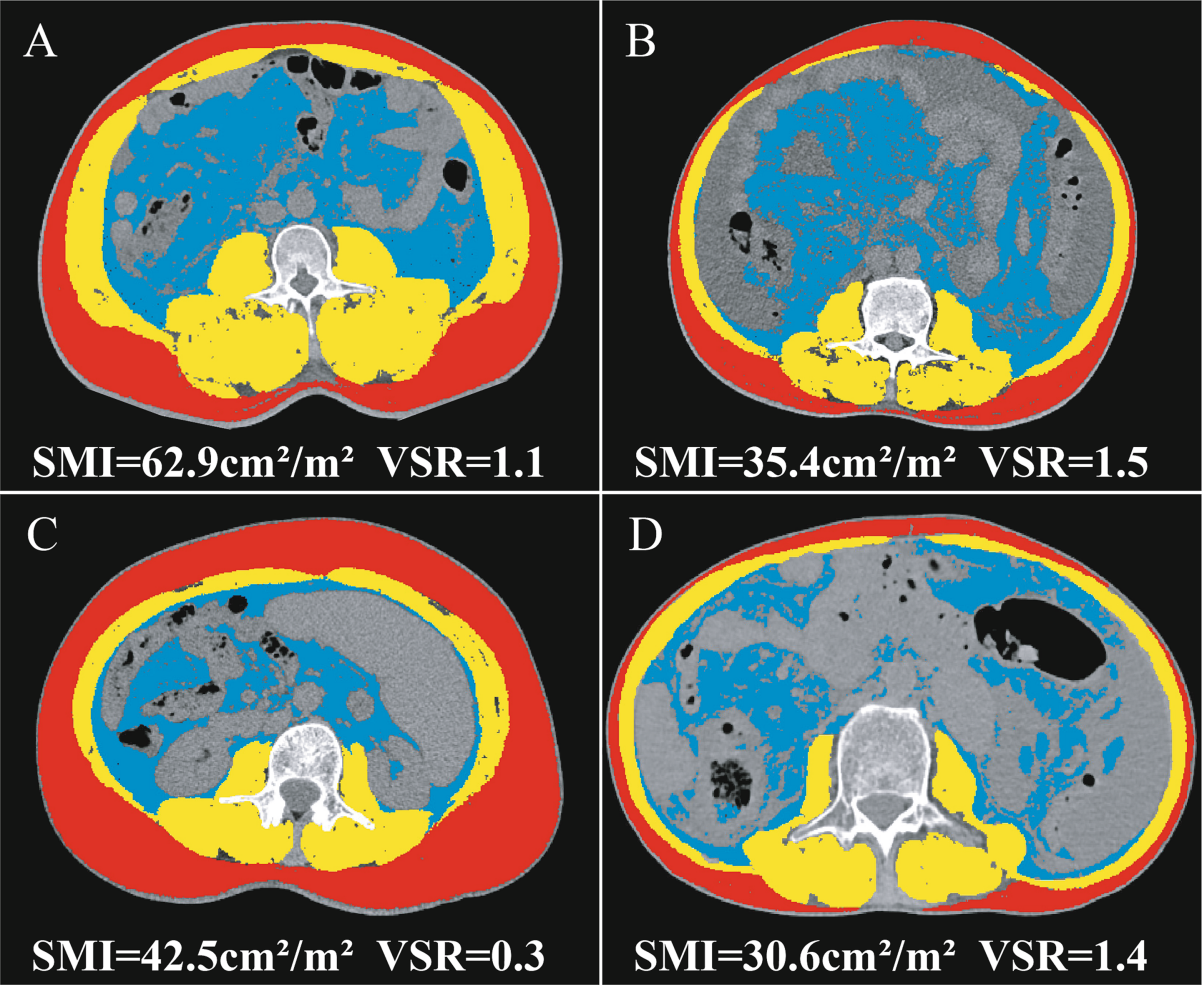
Computed tomography images at the L3 level used to assess low muscle mass and visceral adiposity (LMM-VA). **(A)** Male patient without LMM-VA; **(B)** Male patient with LMM-VA; **(C)** Female patient without LMM-VA; and **(D)** Female patient with LMM-VA. Yellow areas indicate skeletal muscle mass; red areas indicate subcutaneous adipose tissue; and blue areas indicate visceral adipose tissue.

Body composition was evaluated through secondary analysis of abdominal CT scans obtained either for the assessment of cirrhosis-related complications or as part of the pre-TIPS evaluation. Skeletal muscle groups, including the psoas, erector spinae, quadratus lumborum, transversus abdominis, external and internal obliques, and rectus abdominis, as well as VAT and SAT, were quantified at the third lumbar (L3) vertebral level. SAT was defined as adipose tissue located beneath the skin and above the parietal peritoneum, while VAT was defined as intraperitoneal adipose tissue. All image analyses were performed by a trained radiologist (M.S.) who was blinded to clinical and outcome data, using SliceOMatic software (version 5.0; TomoVision, Montreal, Canada). Tissue segmentation was conducted based on standard Hounsfield unit (HU) thresholds: −29 to 150 for skeletal muscle, −150 to −50 for VAT, and −190 to −30 for SAT (10). Cross-sectional areas (cm^2^) were obtained by semi-automated tracing of tissue boundaries, which were manually adjusted to accurately match the actual muscle, visceral, and subcutaneous adipose boundaries on a single CT slice (Figure 1). The software calculated total areas by summing tissue pixels and multiplying by pixel surface area. All measurements were normalized for height (m^2^) to derive the SMI (cm^2^/m^2^), VAT index (VATI, cm^2^/m^2^), and SAT index (SATI, cm^2^/m^2^). The VSR was calculated as VATI divided by SATI. Low muscle mass (LMM) was defined based on previously validated cutoff values for Chinese patients with cirrhosis as SMI < 32.5 cm^2^/m^2^ in females and < 44.7 cm^2^/m^2^ in males (17). Visceral adiposity (VA) was defined according to previously published studies in patients with cirrhosis as a VSR ≥ 1.21 in males and ≥0.48 in females (10). LMM-VA was defined as the coexistence of LMM and VA. In addition, as a supplementary analysis, VA was alternatively defined as BMI of ≥25 kg/m^2^ or VAT area of ≥100 cm^2^ (18).

### 2.3 TIPS placement

All TIPS procedures were performed under local anesthesia by experienced interventional radiologists (S.D., J.Y., and X.T.) according to standard operating procedures. The procedure was performed as follows. After successful puncture of the right internal jugular vein, a transjugular liver access set (RUPS-100; Cook Medical LLC, Bloomington, IN, United States) was advanced over a guidewire into the right hepatic vein. Under fluoroscopic guidance, the hepatic parenchyma was subsequently punctured to gain access to the portal vein, typically at the left or right portal branch, or at the bifurcation when necessary. When collateral veins were observed on pre-TIPS portography, collateral embolization was performed whenever feasible using coils and/or Histoacryl, at the discretion of the interventional team. The portosystemic pressure gradient (PPG) was measured before shunt creation. An 8-mm polytetrafluoroethylene-covered stent endoprosthesis (Viatorr CX Stent; W. L. Gore and Associates Inc., Flagstaff, AZ, United States) was deployed, followed by balloon dilatation to a diameter of 6–8 mm based on the hemodynamic response. The PPG was re-measured to confirm that the target level (≤ 12 mmHg or a reduction of ≥ 50% from baseline) was achieved, thereby ensuring effective portal decompression.

### 2.4 Outcomes and definitions

The primary outcome was all-cause mortality after TIPS. Secondary outcomes included liver-related death and development of new-onset decompensation and further decompensation. New-onset decompensation was defined as the first appearance of ascites or HE not present before the TIPS procedure (Supplementary Figure 1). Further decompensation was defined as the development of a second portal hypertension-driven decompensating event or the recurrence of variceal bleeding, ascites, or HE after TIPS (Supplementary Figure 1) (19). Ascites was diagnosed based on compatible clinical findings and confirmed by ultrasound, CT, or abdominal paracentesis. HE was defined as grade II or higher according to the West Haven criteria (19). The comorbidity burden was assessed using the Cirrhosis Comorbidity (CirCom) score, a scoring system developed specifically for patients with cirrhosis (20) and comprised of nine comorbid conditions including chronic obstructive pulmonary disease, acute myocardial infarction, and peripheral arterial disease, among others.

### 2.5 Statistical analyses

No prospective sample size calculation was performed at study initiation, as all eligible patients were consecutively enrolled in this retrospective cohort. Post-hoc statistical power analysis was conducted using PASS 11.0 (NCSS, LLC. Kaysville, UT, United States) based on sample size, survival proportions and hazard ratio (HR). Using a two-sided alpha level of 0.05, the current sample achieved a statistical power of 84.0% to detect the significant prognostic association between LMM-VA and all-cause mortality, indicating that the sample size was sufficient for evaluating the primary endpoint.

Data were summarized as mean (standard deviation) or median (interquartile range) for continuous variables, depending on whether they followed a normal distribution, and as counts and percentages for categorical variables. Between-group comparisons were performed using the *t*-test or Mann–Whitney *U* test for continuous variables, and the χ^2^ test or Fisher’s exact test for categorical variables, as appropriate. Standardized mean differences (SMDs) were calculated to quantify the balance of baseline covariates before and after inverse probability of treatment weighting (IPTW). An SMD < 0.1 indicated adequate balance between groups. Time zero for time-to-event analyses was defined as the date of the index CT used for body composition assessment. For all-cause mortality, Kaplan–Meier curves with log-rank test and Cox proportional hazards regression were applied, as no patients in our cohort underwent liver transplantation. For secondary outcomes, cumulative incidence function curves with Gray’s test and competing risk regression analyses using the Fine–Gray subdistribution hazard model were performed. Liver-related death was analyzed with non-liver-related death as a competing event, whereas new-onset and further decompensation were analyzed with all-cause death as the competing event. The impact of LMM-VA on the primary and secondary outcomes was evaluated using univariable and multivariable analyses. Established clinical prognostic factors, such as the model for end-stage liver disease (MELD) score and cirrhosis comorbidity score, along with candidate predictors showing *p* < 0.10 in univariable analysis, were entered into multivariable Cox regression or competing risk models. Redundant variables were excluded from the final analysis to minimize potential collinearity. Multiple sensitivity analyses were performed for the primary outcome as follows: (1) The MELD score in the multivariable model was replaced by either the Child–Turcotte–Pugh (CTP) score or CTP class; (2) Multivariable regression model with Firth correction; (3) Multivariable Cox regression model with time-zero redefined as the date of TIPS placement; (4) Multivariable Cox regression model adjusted via IPTW; (5) Multivariable Cox regression model verified by 1,000 bootstrap iterations; and (6) Data-driven sex-specific cut-off values derived from the maximum Youden index were applied to define LMM-VA (Supplementary Table 1). Subgroup analyses were performed according to the prespecified clinical variables of age (<60/≥60 years), sex (male/female), etiology (viral hepatitis/others), and MELD score (<10/≥10). Stratified multivariable Cox regression models were applied, with covariate adjustments modified by excluding the stratification variable within each subgroup to avoid overadjustment. Potential interaction effects were examined by introducing multiplicative interaction terms into the overall models. The association between LMM-VA and all-cause mortality was further evaluated by stratifying patients into four groups based on muscle and adipose tissue abnormalities, namely normal, isolated VA, isolated LMM, and LMM-VA. Different VA criteria, such as BMI ≥ 25 kg/m^2^ or VAT area ≥ 100 cm^2^, were used to replace VSR in defining LMM-VA for predicting all-cause mortality, with the same covariates as in the main analysis adjusted for in the multivariable Cox regression. To assess the incremental prognostic value of LMM-VA, we evaluated the predictive performance of models combining either CTP score or MELD score with LMM-VA. Harrell’s C-index, Brier score and net reclassification improvement were further calculated. All statistical analyses were performed using R software (version 4.4.1; R Foundation for Statistical Computing, Vienna, Austria), with two-tailed *p* < 0.05 considered statistically significant.

## 3 Results

### 3.1 Baseline characteristics

After applying the inclusion and exclusion criteria, a total of 228 patients with cirrhosis who successfully underwent TIPS procedures were included in the study (Supplementary Figure 2). To evaluate potential selection bias, we compared the key baseline characteristics of the included and excluded patients, as shown in Supplementary Table 2. No statistically significant differences were found between the two groups in key baseline variables, including age, sex, liver disease etiology, CTP score, MELD score, CirCom score, and others (all *p* > 0.05). The baseline characteristics of the included patients are summarized in Table 1. Overall, the cohort predominantly consisted of males (55.7%) with a mean age of 55.8 ± 11.1 years. The primary etiology of cirrhosis was viral hepatitis (63.6%), followed by autoimmune liver disease (12.7%) and alcoholic liver disease (7.9%). The proportions of patients with ascites, prior HE, and portal vein thrombosis were 64.5, 3.5, and 36.4%, respectively. A total of 17.5% of patients had a CirCom score ≥ 1. Nearly half of the patients (47.4%) had received previous endoscopic or NSBB therapy. Most patients (59.6%) were classified as CTP class B, with a median CTP score of 7.0 and a median MELD score of 10.0. Of the 228 included patients, 54 (23.7%) were identified as having LMM-VA. Compared with the non-LMM-VA group, the patients with LMM-VA were older, had a lower proportion of viral hepatitis, and were more likely to present with ascites (all *p* < 0.05). They also had a higher frequency of prior endoscopic or NSBB treatment, along with higher CTP scores and neutrophil-to-lymphocyte ratio values (all *p* < 0.05). Regarding body composition, these patients exhibited lower BMI, SMI, and SATI, but higher VSR values (all *p* < 0.001). In addition, SMD were calculated to assess baseline balance before and after IPTW adjustment (Supplementary Table 3).

**Table 1:** Baseline characteristics of patients with cirrhosis undergoing TIPS grouped by low muscle mass and visceral adiposity.

Variable	Overall (*n* = 228)	Non-low muscle mass and visceral adiposity (*n* = 174)	Low muscle mass and visceral adiposity (*n* = 54)	*p* value
Age, mean ± SD, years	55.8 (11.1)	54.6 (10.8)	59.8 (10.9)	0.002
Sex, *n* (%)				0.419
Male	127 (55.7)	100 (57.5)	27 (50.0)	
Female	101 (44.3)	74 (42.5)	27 (50.0)	
Etiology, *n* (%)				0.018
Viral hepatitis	145 (63.6)	119 (68.4)	26 (48.1)	
Alcohol	18 (7.9)	11 (6.3)	7 (13.0)	
Autoimmune	29 (12.7)	17 (9.8)	12 (22.2)	
Others	36 (15.8)	27 (15.5)	9 (16.7)	
Smoking, *n* (%)				0.280
No	171 (75.0)	134 (77.0)	37 (68.5)	
Yes	57 (25.0)	40 (23.0)	17 (31.5)	
Drinking, *n* (%)				0.412
No	184 (80.7)	143 (82.2)	41 (75.9)	
Yes	44 (19.3)	31 (17.8)	13 (24.1)	
Ascites, *n* (%)				0.030
No	81 (35.5)	69 (39.7)	12 (22.2)	
Yes	147 (64.5)	105 (60.3)	42 (77.8)	
Previous hepatic encephalopathy, *n* (%)				0.608
No	220 (96.5)	169 (97.1)	51 (94.4)	
Yes	8 (3.5)	5 (2.9)	3 (5.6)	
Portal vein thrombosis, *n* (%)				1.000
No	145 (63.6)	111 (63.8)	34 (63.0)	
Yes	83 (36.4)	63 (36.2)	20 (37.0)	
CirCom score, *n* (%)				0.104
0	188 (82.5)	139 (79.9)	49 (90.7)	
≥1	40 (17.5)	35 (20.1)	5 (9.3)	
Previous endoscopic or NSBB treatment, *n* (%)				0.013
No	120 (52.6)	100 (57.5)	20 (37.0)	
Yes	108 (47.4)	74 (42.5)	34 (63.0)	
CTP score, median (IQR)	7.0 (6.0, 8.0)	7.0 (6.0, 8.0)	7.5 (6.3, 8.0)	0.011
CTP classification, *n* (%)				0.125
A	80 (35.1)	66 (37.9)	14 (25.9)	
B	136 (59.6)	101 (58.1)	35 (64.8)	
C	12 (5.3)	7 (4.0)	5 (9.3)	
MELD score, median (IQR)	10.0 (8.0, 11.0)	10.0 (8.0, 11.0)	9.5 (8.0, 11.0)	0.830
WBC, median (IQR), 10^9^/L	3.4 (2.3, 5.6)	3.2 (2.4, 4.9)	3.8 (2.1, 6.5)	0.458
Hemoglobin, median (IQR), g/L	80.0 (69.0, 93.3)	80.0 (72.0, 94.0)	81.0 (65.3, 91.8)	0.659
Platelet, median (IQR), 10^9^/L	69.5 (46.0, 95.3)	71.0 (46.3, 95.8)	66.5 (46.3, 93.8)	0.871
Neutrophil-to-lymphocyte ratio, median (IQR)	3.3 (2.2, 5.5)	3.1 (2.1, 5.0)	4.1 (3.1, 7.6)	0.002
INR, median (IQR)	1.2 (1.1, 1.3)	1.2 (1.1, 1.3)	1.2 (1.2, 1.4)	0.290
TBIL, median (IQR), umol/L	24.3 (16.4, 32.4)	24.1 (16.6, 31.7)	25.2 (14.3, 35.0)	0.782
ALT, median (IQR), U/L	19.0 (14.8, 32.0)	19.0 (14.0, 31.0)	20.5 (15.0, 37.8)	0.410
AST, median (IQR), U/L	30.0 (22.0, 43.0)	30.0 (23.0, 41.0)	31.5 (22.0, 51.3)	0.203
Albumin, mean ± SD, g/L	32.7 (5.4)	32.9 (5.4)	32.0 (5.2)	0.311
Creatinine, median (IQR), umol/L	54.3 (45.3, 70.7)	54.2 (45.4, 67.7)	56.6 (43.5, 79.8)	0.378
Serum sodium, mean ± SD, mmol/L	138.5 (3.0)	138.8 (2.9)	137.9 (3.2)	0.054
Blood ammonia, median (IQR), umol/L	59.5 (46.0, 80.0)	61.5 (47.3, 80.8)	57.0 (43.3, 73.0)	0.187
BMI, median (IQR), kg/m^2^	22.5 (20.3, 24.8)	22.9 (20.9, 25.6)	20.4 (19.0, 23.1)	<0.001
SMI, median (IQR), cm^2^/m^2^	41.6 (35.7, 48.5)	44.7 (38.4, 50.9)	31.3 (28.7, 37.6)	<0.001
VATI, median (IQR), cm^2^/m^2^	28.1 (18.1, 40.5)	28.7 (17.7, 40.9)	25.3 (19.2, 38.8)	0.964
SATI, median (IQR), cm^2^/m^2^	32.5 (19.4, 49.1)	37.1 (23.1, 51.3)	22.7 (12.1, 34.3)	<0.001
VSR, median (IQR)	0.9 (0.6, 1.3)	0.9 (0.6, 1.1)	1.4 (0.9, 1.9)	<0.001

Between-group comparisons were performed using the *t*-test or Mann–Whitney U test for continuous variables, and the χ^2^ test or Fisher’s exact test for categorical variables, as appropriate. TIPS, transjugular intrahepatic portosystemic shunt; SD, standard deviation; CirCom, cirrhosis comorbidity; NSBB, non-selective beta-blockers‌; CTP, Child-Turcotte-Pugh; IQR, interquartile range; MELD, model for end-stage liver disease; WBC, white blood cell; INR, international normalized ratio; TBIL, total bilirubin; ALT, alanine aminotransferase; AST, aspartate aminotransferase; BMI, body mass index; SMI, skeletal muscle index; VATI, visceral adipose tissue index; SATI, subcutaneous adipose tissue index; VSR, visceral to subcutaneous ratio.

### 3.2 Impact of LMM-VA on intraoperative and postoperative outcomes

Intraoperative and postoperative outcomes are summarized in Table 2. For intraoperative outcomes, 76.3% of patients underwent shunting via the left portal vein branch, followed by 14.5% via the right portal vein branch and 9.2% via the portal vein bifurcation. The vast majority of patients (90.4%) underwent collateral embolization before TIPS stent placement. The median pre-TIPS PPG was 35.0 mmHg, which decreased to 12.0 mmHg after stent placement, with a median reduction of 23.0 mmHg. With an 8-mm covered stent, 93.4% of patients achieved the target PPG level (≤ 12 mmHg or a reduction of ≥ 50% from baseline). There were no significant differences in any intraoperative outcomes between patients with and without LMM-VA. By the end of follow-up (median duration of 29.2 [13.4–46.1] months), 26 patients (11.4%) experienced all-cause death, 20 (76.9%) of whom died from liver-related causes. The incidence of new-onset decompensation was 26.8% of patients, including new-onset ascites in 5.3% and new-onset HE in 21.5%. Further decompensation occurred in 38.6% of patients, with recurrent variceal bleeding in 7.9%, new or recurrent ascites in 11.0%, and new or recurrent HE in 22.4%. Patients with LMM-VA had significantly higher rates of all-cause and liver-related death compared with those without LMM-VA; however, no significant differences were observed between the two groups in the incidence of new-onset or further decompensation events.

**Table 2:** Comparison of intraoperative and postoperative outcomes in patients with cirrhosis undergoing TIPS grouped by low muscle mass and visceral adiposity.

Outcome	Overall (*n* = 228)	Non-low muscle mass and visceral adiposity (*n* = 174)	Low muscle mass and visceral adiposity (*n* = 54)	*p* value
Intraoperative outcome				
Shunt position, *n* (%)				0.262
Left portal vein	174 (76.3)	135 (77.6)	39 (72.2)	
Right portal vein	33 (14.5)	26 (14.9)	7 (13.0)	
Portal vein bifurcation	21 (9.2)	13 (7.5)	8 (14.8)	
Collateral embolization	206 (90.4)	157 (90.2)	49 (90.7)	1.000
PPG (before TIPS stent), median (IQR), mmHg	35.0 (31.0, 40.0)	34.0 (31.0, 39.8)	35.0 (30.3, 42.8)	0.343
PPG (after TIPS stent), median (IQR), mmHg	12.0 (9.0, 15.0)	12.0 (9.0, 15.0)	12.0 (9.3, 15.0)	0.445
PPG reduction, median (IQR), mmHg	23.0 (19.0, 27.0)	23.0 (19.0, 27.0)	23.0 (20.0, 28.0)	0.441
Achievement of target PPG reduction^a^, *n* (%)	213 (93.4)	160 (92.0)	53 (98.1)	0.197
Postoperative outcome				
Follow-up time, median (IQR), months	29.2 (13.4, 46.1)	30.5 (13.1, 46.7)	26.1 (15.1, 37.6)	0.120
All-cause Death, *n* (%)	26 (11.4)	14 (8.0)	12 (22.2)	0.009
Liver-related death, *n* (%)	20 (8.8)	10 (5.7)	10 (18.5)	0.009
New-onset decompensation, *n* (%)	61 (26.8)	47 (27.0)	14 (25.9)	1.000
New-onset ascites, *n* (%)	12 (5.3)	11 (6.3)	1 (1.9)	0.349
New-onset hepatic encephalopathy, *n* (%)	49 (21.5)	36 (20.7)	13 (24.1)	0.734
Further decompensation, *n* (%)	88 (38.6)	68 (39.1)	20 (37.0)	0.913
Recurrent variceal bleeding, *n* (%)	18 (7.9)	14 (8.0)	4 (7.4)	1.000
New or recurrent ascites, *n* (%)	25 (11.0)	21 (12.1)	4 (7.4)	0.479
New or recurrent hepatic encephalopathy, *n* (%)	51 (22.4)	37 (21.3)	14 (25.9)	0.595

Between-group comparisons were performed using the *t*-test or Mann–Whitney U test for continuous variables, and the χ^2^ test or Fisher’s exact test for categorical variables, as appropriate. TIPS, transjugular intrahepatic portosystemic shunt; PPG, portal pressure gradient; IQR, interquartile range.

a Postoperative PPG <12 mmHg or a > 50% relative reduction from the baseline.

### 3.3 Associations between LMM-VA and post-TIPS clinical outcomes

**Figure 2:**
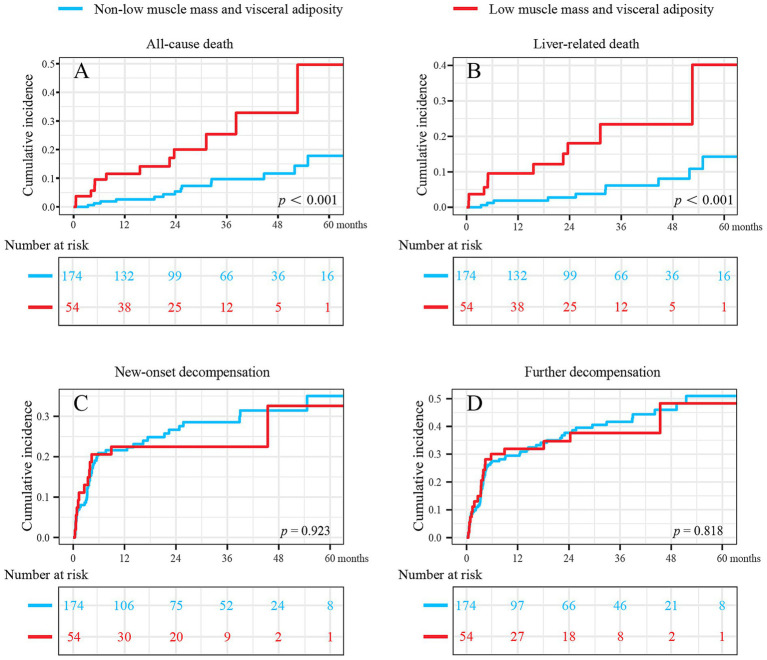
Kaplan–Meier and cumulative incidence function curves comparing the cumulative risk of clinical outcomes between the two groups. **(A)** Kaplan–Meier curves illustrating the cumulative incidence of all-cause death; **(B)** Cumulative incidence function curves for liver-related death, with non–liver-related death as a competing event; **(C)** Cumulative incidence function curves for new-onset decompensation, with all-cause death as a competing event; and **(D)** Cumulative incidence function curves for further decompensation, with all-cause death as a competing event.

As shown in Figure 2, throughout the follow-up period, patients with LMM-VA exhibited a significantly higher cumulative incidence of all-cause death (log-rank test *p* < 0.001) and liver-related death (Gray’s test *p* < 0.001) compared with those without LMM-VA. By contrast, no significant differences were observed between the two groups in the cumulative incidence of new-onset decompensation (Gray’s test *p* = 0.923) or further decompensation (Gray’s test *p* = 0.818).

**Figure 3:**
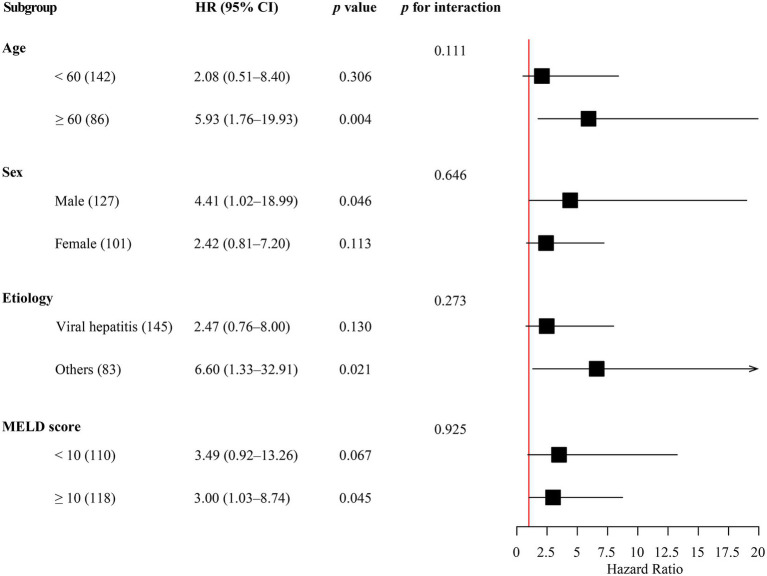
Forest plot showing the association between LMM-VA and all-cause mortality across different subgroups. LMM-VA, low muscle mass and visceral adiposity.

In univariable Cox regression analysis, patients with LMM-VA had a significantly higher risk of all-cause death compared with those without LMM-VA (HR: 3.62, 95% confidence interval [CI]: 1.65–7.91, *p* = 0.001; Table 3). This association remained significant after adjustment for age, CirCom score, MELD score, hemoglobin, serum sodium, pre-TIPS PPG and PPG reduction (HR: 3.21, 95% CI: 1.32–7.80, *p* = 0.010; Table 3). In sensitivity analyses, similar results were obtained when the MELD score was replaced in the multivariable model with either the CTP score (HR: 3.36, 95% CI: 1.37–8.23, *p* = 0.008) or the CTP class (HR: 3.45, 95% CI: 1.39–8.55, *p* = 0.008). Consistent outcomes were also observed in the other five sensitivity analyses (Supplementary Table 4). Subgroup analyses across different age strata, sex, etiologies, and MELD score strata demonstrated consistent results (all interaction *p* > 0.1; Figure 3).

**Table 3:** Univariable and multivariable Cox regression for predictors of all-cause death in patients with cirrhosis undergoing TIPS.

Variable	Univariable analysis		Multivariable analysis	
	HR (95% CI)	*p* value	HR (95% CI)	*p* value
Age, years	1.56 (1.02–2.38)	0.041	1.28 (0.85–1.93)	0.236
Sex				
Male	1.00 (reference)			
Female	1.33 (0.61–2.88)	0.470		
Etiology				
Viral hepatitis	1.00 (reference)			
Alcohol	1.09 (0.25–4.75)	0.904		
Autoimmune	0.60 (0.14–2.61)	0.497		
Others	1.05 (0.39–2.85)	0.927		
Smoking	1.06 (0.42–2.64)	0.908		
Drinking	1.89 (0.79–4.53)	0.152		
Ascites	1.52 (0.66–3.50)	0.329		
Previous hepatic encephalopathy	NC^a^	0.997		
Portal vein thrombosis	0.88 (0.39–1.97)	0.751		
CirCom score				
0	1.00 (reference)		1.00 (reference)	
≥1	1.30 (0.52–3.23)	0.579	2.04 (0.77–5.42)	0.153
Previous endoscopic or NSBB treatment	1.00 (0.46–2.16)	0.999		
CTP score	1.22 (0.87–1.73)	0.249		
CTP classification				
A	1.00 (reference)			
B	1.93 (0.80–4.66)	0.143		
C	1.13 (0.14–9.23)	0.906		
MELD score	1.12 (0.75–1.66)	0.576	1.16 (0.77–1.74)	0.484
Low muscle mass and visceral adiposity	3.62 (1.65–7.91)	0.001	3.21 (1.32–7.80)	0.010
WBC, 10^9^/L	1.12 (0.79–1.59)	0.516		
Hemoglobin, g/L	0.62 (0.40–0.97)	0.037	0.71 (0.45–1.11)	0.135
Platelet, 10^9^/L	1.10 (0.83–1.47)	0.505		
Neutrophil-to-lymphocyte ratio	1.25 (0.89–1.76)	0.202		
INR	1.03 (0.70–1.51)	0.874		
TBIL, umol/L	1.04 (0.75–1.46)	0.801		
ALT, U/L	0.98 (0.70–1.36)	0.894		
AST, U/L	1.15 (0.87–1.36)	0.321		
Albumin, g/L	0.80 (0.54–1.19)	0.275		
Creatinine, umol/L	1.35 (0.95–1.92)	0.096^b^		
Serum sodium, mmol/L	0.71 (0.50–1.02)	0.063	0.78 (0.53–1.14)	0.194
Blood ammonia, umol/L	1.29 (0.92–1.81)	0.141		
BMI, kg/m^2^	0.75 (0.49–1.15)	0.185		
SMI, cm^2^/m^2^	0.67 (0.47–0.95)	0.023^c^		
VATI, cm^2^/m^2^	0.70 (0.43–1.12)	0.137		
SATI, cm^2^/m^2^	0.74 (0.47–1.17)	0.203		
VSR	1.04 (0.69–1.58)	0.850		
Shunt position, *n* (%)				
Left portal vein	1.00 (reference)			
Right portal vein	0.63 (0.15–2.71)	0.538		
Portal vein bifurcation	2.04 (0.70–5.99)	0.193		
Collateral embolization	0.46 (0.17–1.21)	0.114		
PPG (before TIPS stent), mmHg	0.67 (0.44–1.00)	0.052	0.71 (0.38–1.32)	0.278
PPG (after TIPS stent), mmHg	0.89 (0.60–1.31)	0.558		
PPG reduction, mmHg	0.67 (0.44–1.02)	0.064	0.85 (0.44–1.65)	0.639

TIPS, transjugular intrahepatic portosystemic shunt; HR, hazard ratio; CI, confidence interval; NC, not calculable; CirCom, cirrhosis comorbidity; NSBB, non-selective beta-blockers‌; CTP, Child-Turcotte-Pugh; MELD, model for end-stage liver disease; WBC, white blood cell; INR, international normalized ratio; TBIL, total bilirubin; ALT, alanine aminotransferase; AST, aspartate aminotransferase; BMI, body mass index; SMI, skeletal muscle index; VATI, visceral adipose tissue index; SATI, subcutaneous adipose tissue index; VSR, the visceral to subcutaneous ratio; PPG, portal pressure gradient.

a Hazard ratio and confidence interval not estimable due to sparse data.

b Creatinine was not included in the multivariate analysis because it is a component of the MELD score.

c SMI was not included in the multivariate analysis because it was a component of the definition of low muscle mass and visceral adiposity.

All patients were stratified into four groups according to muscle and adipose tissue abnormalities: normal (*n* = 72; 31.6%), isolated VA (*n* = 78; 34.2%), isolated LMM (*n* = 24; 10.5%), and LMM-VA (*n* = 54; 23.7%). Compared with the normal group, patients with LMM-VA exhibited a significantly higher cumulative incidence of all-cause death (log-rank *p* = 0.036), whereas no significant difference was observed for those with isolated VA (log-rank *p* = 0.675) or isolated LMM (log-rank *p* = 0.714) (Supplementary Figure 3). Similarly, compared with the normal group, only patients with LMM-VA showed a significant association with all-cause death in both univariable (HR: 4.12, 95% CI: 1.44–11.76, *p* = 0.008) and multivariable Cox regression analyses adjusted for the same covariates as in the main analysis (HR: 4.14, 95% CI: 1.28–13.38, *p* = 0.018) (Supplementary Table 5). Furthermore, the combination of LMM-VA with either CTP or MELD score achieved moderate discriminative ability and satisfactory calibration, and significantly improved the net reclassification performance of the original scoring systems (Supplementary Table 6).

For liver-related death, LMM-VA also demonstrated an independent prognostic value, with a subdistribution (s)HR of 4.25 (95% CI: 1.80–10.03, *p* = 0.001) in the univariable competing risk model and 4.18 (95% CI: 1.69–10.30, *p* = 0.002) in the multivariable model after adjustment for CirCom score, CTP classification, hemoglobin, creatinine, pre-TIPS PPG and PPG reduction (Supplementary Table 7). By contrast, LMM-VA was not independently associated with new-onset decompensation (sHR: 0.91, 95% CI: 0.49–1.68, *p* = 0.754) or further decompensation (sHR: 0.98, 95% CI: 0.60–1.62, *p* = 0.940).

### 3.4 Comparison of different visceral obesity criteria: VSR, BMI, and VAT area

Finally, we evaluated the predictive value of LMM-VA for all-cause death when the VSR criterion was replaced by alternative definitions, including a VAT area ≥ 100 cm^2^ or BMI ≥ 25.0 kg/m^2^. Kaplan–Meier analyses showed no significant differences in the cumulative incidence of all-cause death between the two groups, regardless of whether LMM-VA was defined by LMM-VA_VAT (log-rank test *p* = 0.600) or LMM-VA_BMI (log-rank test *p* = 0.810) (Supplementary Figure 4). Consistent with the main analysis, after adjusting for age, CirCom score, MELD score, hemoglobin, serum sodium, pre-TIPS PPG and PPG reduction, no statistically significant association with all-cause death was observed when LMM-VA was defined by either VAT area ≥ 100 cm^2^ (HR: 1.00, 95% CI: 0.26–3.83, *p* = 0.995) or BMI ≥ 25.0 kg/m^2^ (HR: 0.59, 95% CI: 0.07–5.18, *p* = 0.638) (Supplementary Figure 4).

## 4 Discussion

In this retrospective cohort study of patients with cirrhosis undergoing TIPS for variceal bleeding, we observed that 66.4% of patients had muscle and adipose tissue abnormalities as defined by CT-quantified body composition, and nearly one-quarter were classified as having the combined abnormality of LMM-VA. During a median follow-up of approximately 30 months, patients with LMM-VA exhibited a markedly higher cumulative all-cause mortality than those without LMM-VA, with cumulative mortality exceeding 22% in the LMM-VA group compared with only 8% in the non-LMM-VA group. Multivariable Cox regression analysis showed that, compared with either the non-LMM-VA or normal group, patients with LMM-VA had more than a three- to fourfold higher risk of all-cause death after adjustment for age, CirCom score, MELD score, hemoglobin, serum sodium, pre-TIPS PPG, and PPG reduction. Sensitivity and subgroup analyses focusing on different age, sex, etiology, and MELD score categories confirmed the robustness of the primary outcome. Notably, unlike LMM-VA defined by the VSR-based criteria, LMM-VA defined by either a VAT area of ≥ 100 cm^2^ or a BMI of ≥ 25.0 kg/m^2^ showed no significant predictive value for all-cause death. For secondary outcomes, patients with LMM-VA also exhibited an approximately fourfold higher risk of liver-related mortality compared with those without LMM-VA, even when a more conservative competing risk model was applied. However, LMM-VA was not independently associated with new-onset or further decompensation events.

Although obesity is commonly assessed using BMI in clinical practice due to its convenience, studies evaluating the association between obesity and clinical outcomes in patients with cirrhosis have yielded highly inconsistent results. Some studies have identified obesity as a risk factor for mortality (21), while others have garnered evidence that suggests a protective effect (22, 23) or no significant association (24). These discrepancies may stem from the dynamic changes in fluid retention, sex- and race-related differences in body composition (e.g., VAT, SAT, and skeletal muscle) and the use of varying criteria to define obesity across studies (e.g., BMI ≥ 30 kg/m^2^, ≥ 25 kg/m2 or 27.5 kg/m^2^ in Asian populations) (25). BMI is a crude estimate of obesity, as it encompasses not only fat mass but that of muscle, bone, and visceral organs. In this study, patients with LMM-VA had a significantly lower BMI but a higher proportion of ascites compared with those without LMM-VA, highlighting the unreliability of BMI as a marker of obesity. In a cohort of 300 patients undergoing elective TIPS, BMI was not found to be associated with 1-year post-procedural adverse outcomes (26). A recent meta-analysis including 11 studies with a total of 3,274 patients with cirrhosis also found no significant association between BMI and all-cause mortality beyond 6 months of follow-up (15), which is consistent with our findings. Given the limitations of BMI, cross-sectional CT imaging has emerged as the gold standard for noninvasive assessment of body composition. It enables the objective evaluation of skeletal muscle, visceral fat, and subcutaneous fat distribution to reflect nutritional and metabolic status, using quantitative software with standardized HU thresholds for tissue demarcation (10). Although prior studies have defined VA in patients with sarcopenia and cirrhosis using a CT-quantified VAT area of ≥ 100 cm^2^, this approach did not adjust for sex or body size and did not account for the prognostic impact of SAT. Previous research has demonstrated that the SATI is significantly associated with long-term mortality in patients with cirrhosis (27). Additionally, body fat distribution may play a more crucial role than total body adiposity, as previous studies have shown that VATI is positively associated, whereas SATI is inversely associated, with adverse clinical outcomes in patients with cirrhosis (14, 15, 27). Hence, the relative distribution of adipose tissue, quantified by the VSR rather than the absolute area of fat depots or simple anthropometric indices, may serve as a more comprehensive and sensitive indicator of VA.

In recent years, sarcopenia has been reported to be independently associated with adverse outcomes in patients with cirrhosis, regardless of whether they underwent TIPS (4, 7, 28); however, these findings remain controversial. A cohort study including 146 patients with non-alcoholic steatohepatitis cirrhosis found that sarcopenia was not associated with increased 1-year mortality or rehospitalization following liver transplantation (29). Similarly, a prospective study reported that sarcopenia was not associated with higher rates of hospital admissions or death in patients with cirrhosis and refractory ascites (30). Another study of adult patients who underwent TIPS for refractory ascites found that sarcopenia was not associated with *de novo* HE or increased mortality during a median follow-up of 14.2 months (9). These inconsistencies may indicate that the prognostic value of sarcopenia could be confounded by other coexisting factors. A retrospective study provided evidence that patients with osteosarcopenia, but not those with sarcopenia alone, had significantly lower survival rates than those without musculoskeletal abnormalities (31). A prospective study yielded similar findings in that reduced bone mineral density, rather than sarcopenia, was associated with impaired 12-month survival after TIPS (8). Furthermore, a prospective cohort study demonstrated that frailty, but not sarcopenia, was independently associated with an increased risk of liver-related mortality and decompensation after adjustment for confounders (32), which was also confirmed in another prospective study (33). Two liver transplantation cohorts revealed that sarcopenic visceral obesity, but not sarcopenia, was significantly associated with both pre-transplant waitlist mortality and post-transplant mortality (10, 11). However, the relationship between LMM-VA and clinical outcomes after TIPS in patients with cirrhosis has not been investigated previously. Our findings indicate that patients with LMM-VA, but not those with isolated LMM or VA, are at a significantly higher risk of long-term mortality after TIPS, whereas no association was observed with decompensation events. Interestingly, a previous TIPS cohort study reported similar findings, showing that relative sarcopenia with excess adiposity—defined as the lowest quartile of muscle area as a percentage of total soft tissue area—was a significant predictor of poorer survival, but was not associated with the HE episodes (34).

Abnormal adipose tissue distribution and reduced muscle mass may form a vicious cycle through mutual interaction (12). Sarcopenia leads to decreased physical activity, resulting in reduced energy expenditure and an increased risk of VA. Conversely, the loss of subcutaneous fat and accumulation of visceral fat suppress adiponectin production and induce chronic inflammation within muscle tissue, thereby promoting the development of sarcopenia (12, 16). In our study, the prevalence of LMM-VA was 23.7% among patients with cirrhosis who underwent TIPS. Previous reports have shown that the prevalence of sarcopenic obesity ranges from 2 to 42%, depending on disease stage and the criteria used for the diagnoses of both sarcopenia and obesity (12). Patients with cirrhosis have a high prevalence of malnutrition, particularly those with decompensated cirrhosis (35). Sarcopenia combined with VA may represent a more severe phenotype of malnutrition, predisposing patients to a higher risk of local and systemic infections as well as increased mortality (36). Moreover, patients with sarcopenic obesity have been shown to be more physically vulnerable, with nearly a fourfold higher risk of frailty, which itself is significantly associated with increased mortality, independent of sarcopenia (32, 33, 37). Consistent with these findings, our study demonstrated that LMM-VA in cirrhosis was independently associated with both all-cause and liver-related mortality following TIPS, even after adjusting for age, comorbid burden, severity of liver dysfunction, and portal vein pressure. Interestingly, we failed to identify an independent association of LMM-VA with new-onset or further hepatic decompensation. From the pathophysiological perspective, preoperative LMM-VA is essentially a systemic nutritional and metabolic disorder, rather than a direct driving factor for progressive liver injury. This abnormal body composition status cannot directly aggravate liver fibrosis, deteriorate hepatocellular function, or accelerate the occurrence of typical liver-related decompensation events including refractory ascites, variceal bleeding and HE. In contrast, patients with this adverse body composition usually present with chronic low-grade inflammation, impaired immune defense, reduced physical reserve and poor surgical stress tolerance, which may be the core causes contributing to higher long-term mortality risk (12).

This finding has important clinical implications. First among them is preoperative risk stratification and shared decision-making. Preoperative assessment of LMM-VA status could refine risk stratification for patients undergoing TIPS. Given that patients with LMM-VA exhibit significantly higher post-procedural mortality and may derive reduced clinical benefit from the procedure, these individuals warrant enhanced preoperative risk communication. This includes explicitly discussing the elevated mortality risk with the patient, the potential need for closer monitoring by care staff, and individualizing the risk–benefit balance of TIPS candidacy. It is important to emphasize that LMM-VA should not be considered an absolute contraindication to TIPS; rather, it should serve as a marker to trigger intensified peri-procedural care. Notably, TIPS itself may induce favorable changes in body composition over time, including improvements in muscle mass and reductions in VAT, which could potentially reverse LMM-VA in some patients (13). Second among these are targeted preoperative optimization strategies. For patients undergoing elective TIPS who screen positive for LMM-VA, preoperative interventions aimed at reversing LMM and VA are clinically warranted to mitigate post-procedural risk. Evidence-based strategies include: (1) Ensuring adequate protein intake (1.2–1.5 g/kg/day) to support muscle anabolism; (2) Minimizing prolonged fasting periods to avoid further muscle catabolism; and (3) Implementing structured multicomponent exercise programs combining aerobic, resistance, and flexibility training, which have been shown to increase muscle mass and ameliorate VA in cirrhotic patients (12). Third among these is post-procedural follow-up planning. Patients with pre-procedural LMM-VA may benefit from intensified post-TIPS surveillance, including more frequent clinical assessments, nutritional counseling, and body composition monitoring. This proactive approach aims to identify early signs of disease progression, allowing for timely interventions to optimize long-term clinical outcomes.

This study had several limitations. First, the single-center retrospective design, absence of external validation, and patient exclusion based on CT image availability may introduce selection bias and limit the generalizability of our findings. Nevertheless, we have attempted to counteract this limitation by recruiting consecutive patients, implementing rigorous confounder adjustment, conducting bootstrap internal validation, and comparing core baseline clinical characteristics between included and excluded subjects.

Second, although we conducted multivariable adjustment and various sensitivity analyses in this study, residual confounding cannot be fully ruled out. LMM-VA may only act as an indirect marker reflecting liver disease severity, and a definite causal relationship cannot be established. Further prospective cohort studies are warranted to validate our findings.

Third, although post-hoc power analysis confirmed adequate statistical power (84.0%) for detecting the prognostic effect of LMM-VA, the number of endpoint events in this study remained relatively limited, which may potentially introduce a minor risk of overfitting. To mitigate such bias, we have performed sensitivity analyses using penalized regression and bootstrapping methods. Despite the satisfactory overall statistical power, the stability and generalizability of our findings should still be interpreted with caution.

Fourth, the assessment of LMM-VA requires CT imaging, which limits its use as a primary tool for detecting LMM and VA due to cost and radiation exposure. However, CT scans are commonly performed in patients with cirrhosis for other clinical purposes, such as evaluating cirrhosis-related complications, hepatocellular carcinoma surveillance, or pre-TIPS assessment. Therefore, the secondary use of these scans increases the efficiency of resource utilization. In addition, postoperative longitudinal CT data were not included, preventing dynamic assessment of changes in muscle and adipose tissue composition, which have been shown in previous studies and may further refine the identification of high-risk subgroups.

Fifth, body composition measurements were performed by a single radiologist who was blinded to clinical outcomes. The absence of reproducibility evaluation for image segmentation may lead to potential measurement bias.

Sixth, the predominance of viral etiologies of cirrhosis (63.6%) in our cohort limits the generalizability of our findings to Western populations, where alcohol-related liver disease or MASLD is more prevalent, particularly considering that sarcopenia and visceral obesity have been reported to be more common among patients with those types of liver disease (6, 38). Finally, the adopted SMI and VSR cut-off values may not be fully generalizable to other populations. Future prospective multicenter studies involving international cohorts that consider a broader range of confounders and postoperative body composition changes are warranted to confirm our findings.

## 5 Conclusion

In conclusion, LMM-VA is prevalent among patients with cirrhosis undergoing TIPS and is significantly associated with an increased risk of both overall and liver-related mortality compared with those without LMM-VA. LMM-VA may serve as a valuable prognostic indicator before TIPS. We propose using the relative distribution of adipose tissue, quantified by CT-based VSR, rather than the absolute amount of visceral fat or BMI, as a more informative and clinically meaningful approach to define visceral obesity and to objectively identify patients at risk for suboptimal post-TIPS outcomes.

## Data Availability

The raw data supporting the conclusions of this article will be made available by the authors, without undue reservation.
