# Supplementary material for: Prognostic impact of low muscle mass and visceral adiposity in patients with cirrhosis undergoing transjugular intrahepatic portosystemic shunt: a retrospective cohort study
Source: Front Nutr. 2026 Jul 2;13:1817771. doi: 10.3389/fnut.2026.1817771 (PMC13372622; doi:10.3389/fnut.2026.1817771)
Supplement: Supplementary file 1 [file Table_1.DOCX]

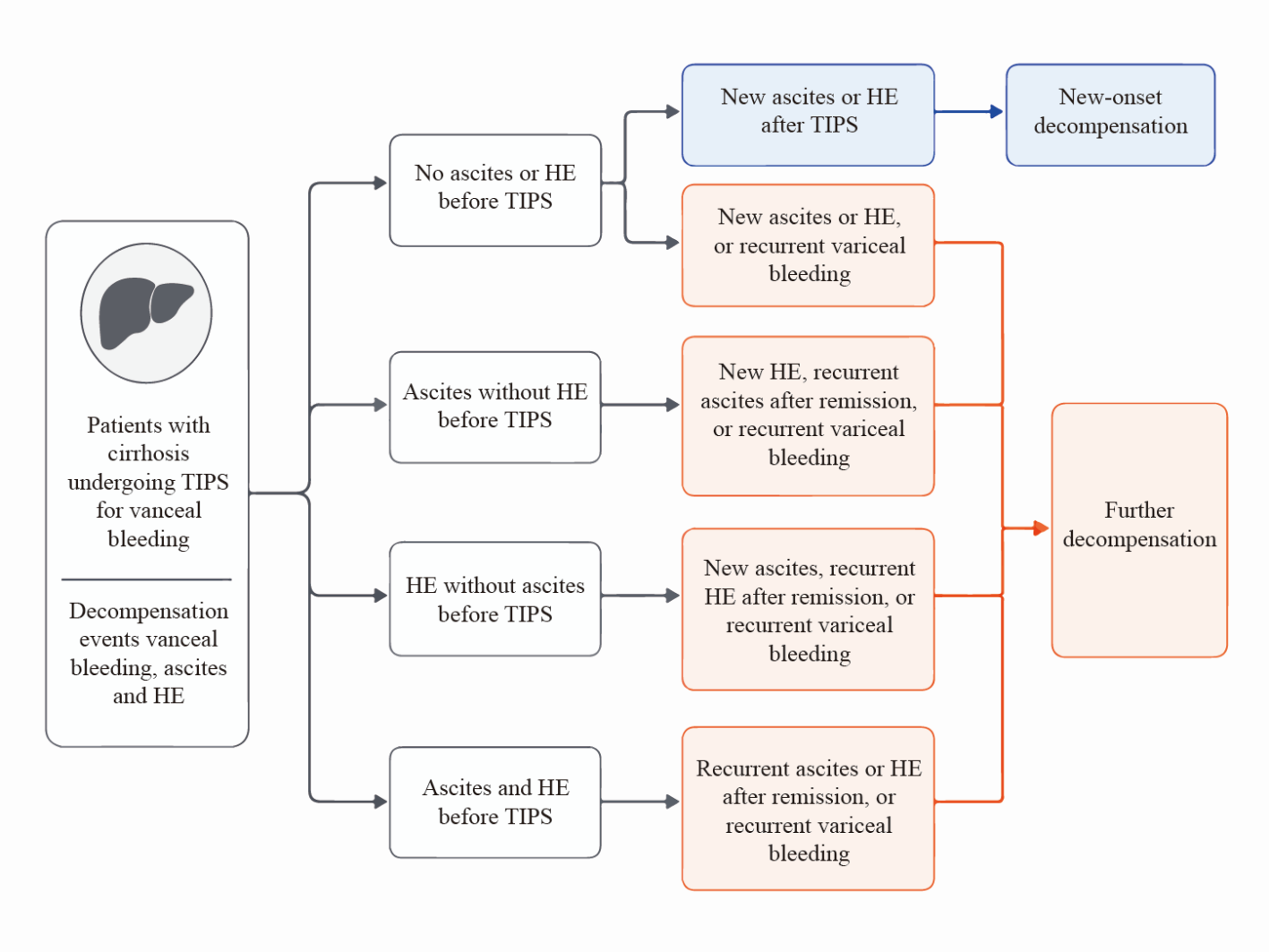


**Supplementary Figure 1.** Schematic illustration of definitions for new-onset decompensation and further decompensation. TIPS, transjugular intrahepatic portosystemic shunt; HE, hepatic encephalopathy.


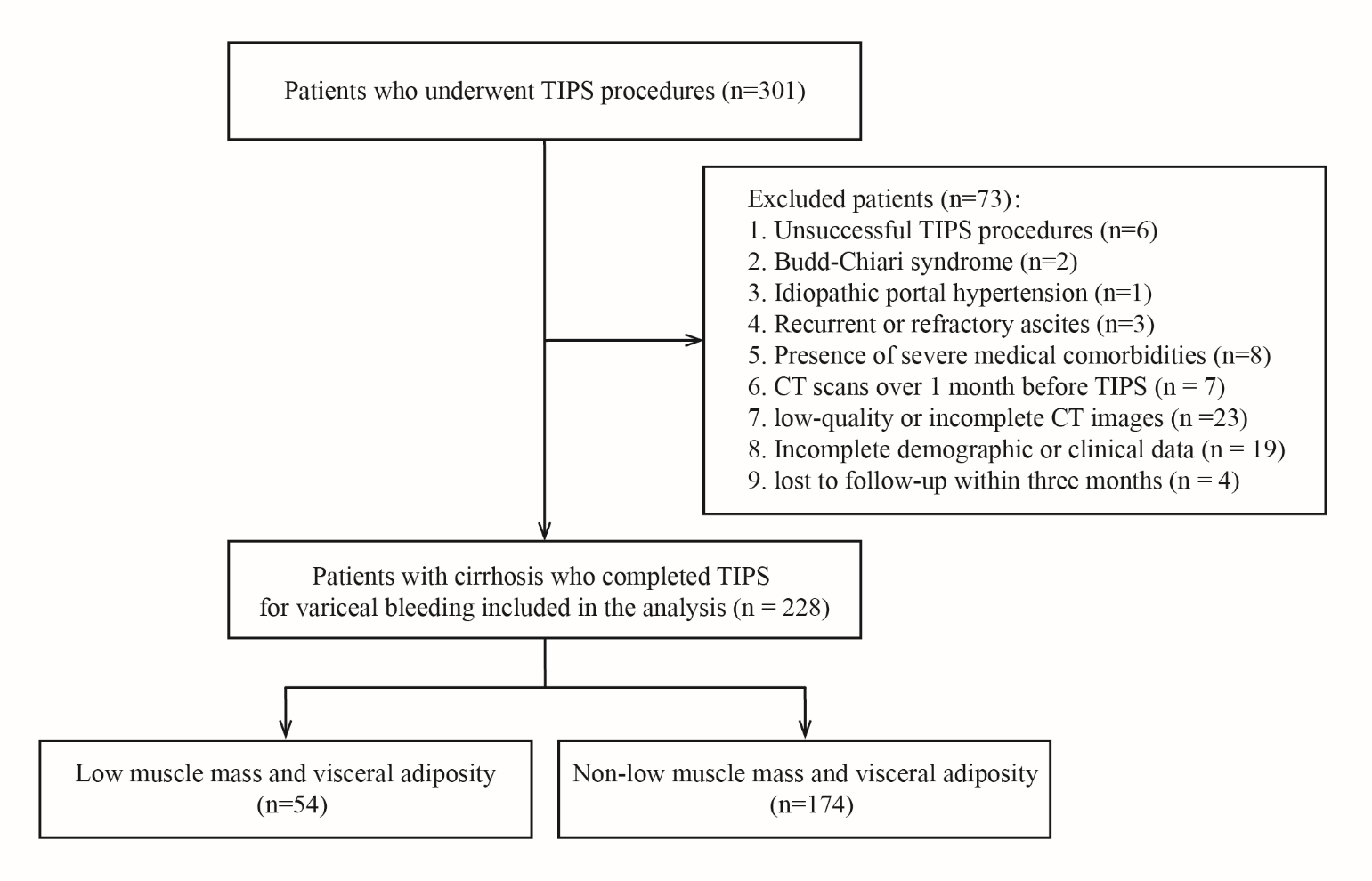


**Supplementary Figure 2.** Flowchart of the patients included in the study.


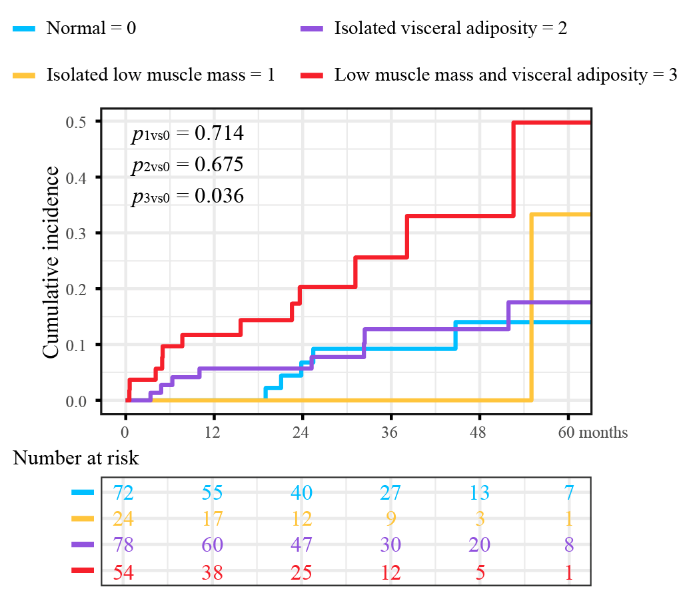


**Supplementary Figure 3.** Kaplan–Meier curves illustrating the cumulative incidence of all-cause death stratified by the presence of low muscle mass and visceral adiposity.


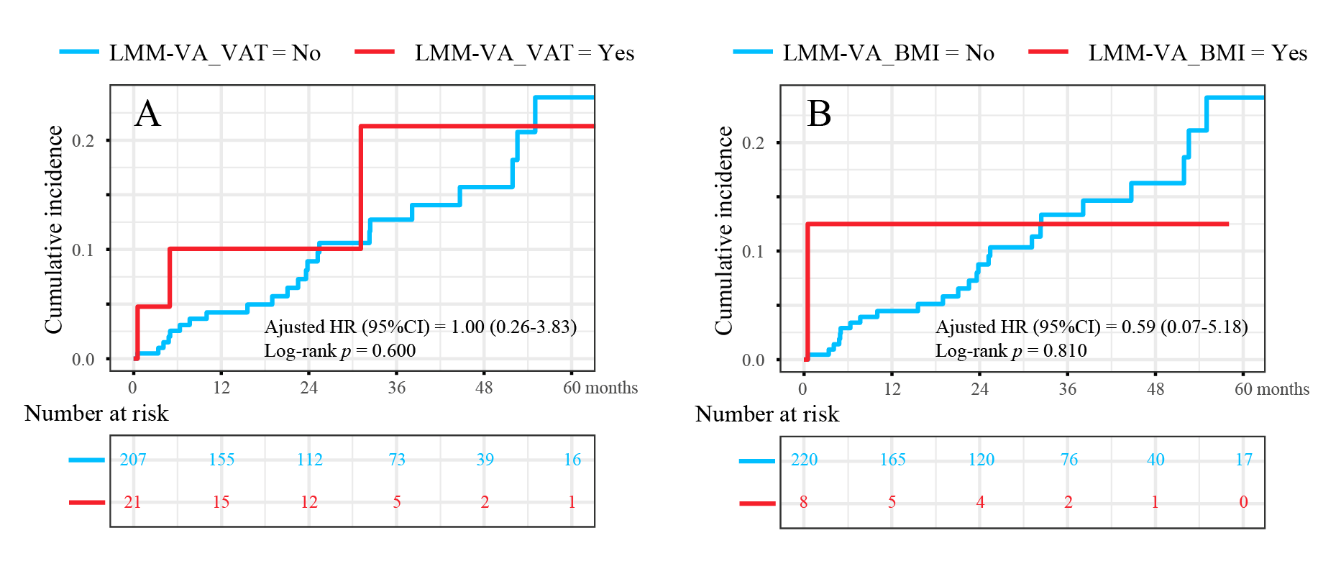


**Supplementary Figure 4.** Kaplan–Meier curves illustrating the cumulative incidence of all-cause death based on different definitions of low muscle mass and visceral adiposity (LMM-VA). (A) LMM-VA defined by visceral adipose tissue area ≥ 100 cm²; (B) LMM-VA defined by body mass index ≥ 25.0 kg/m². LMM-VA, low muscle mass and visceral adiposity; VAT, visceral adipose tissue; BMI, body mass index; HR, hazard ratio.

**Supplementary Table 1.** Data-driven cut-off values for defining low muscle mass and visceral adiposity based on the maximum Youden index.

| **Gender** | **Variable** | **Youden index** | **Cutoff value** |
| --- | --- | --- | --- |
| Male | SMI | 0.45 | 49.31 |
| Male | VSR | 0.37 | 1.40 |
| Female | SMI | 0.43 | 28.04 |
| Female | VSR | 0.06 | 0.86 |

SMI, skeletal muscle index; VSR, visceral to subcutaneous ratio.

**Supplementary Table 2.** Comparison of key baseline characteristics between included and excluded groups.

| **Variable** | **Included group (n=228)** | **Excluded group (n=73)** | ***p* value** |
| --- | --- | --- | --- |
| Age, median (IQR), years | 56.0 (49.0, 64.0) | 56.0 (48.0, 66.0) | 0.877 |
| Sex, n (%) |  |  | 1.000 |
| Male | 127 (55.7) | 41 (56.2) |  |
| Female | 101 (44.3) | 32 (43.8) |  |
| Etiology, n (%) | |  | 0.344 |
| Viral hepatitis | 145 (63.6) | 44 (60.3) |  |
| Alcohol | 18 (7.9) | 11 (15.1) |  |
| Autoimmune | 29 (12.7) | 9 (12.3) |  |
| Others | 36 (15.8) | 9 (12.3) |  |
| Smoking, n (%) | |  | 0.345 |
| No | 171 (75.0) | 59 (80.8) |  |
| Yes | 57 (25.0) | 14 (19.2) |  |
| Drinking, n (%) | |  | 0.866 |
| No | 184 (80.7) | 58 (79.5) |  |
| Yes | 44 (19.3) | 15 (20.5) |  |
| Portal vein thrombosis, n (%) | | | 0.270 |
| No | 145 (63.6) | 41 (56.2) |  |
| Yes | 83 (36.4) | 32 (43.8) |  |
| CirCom score, n (%) | |  | 0.722 |
| 0 | 188 (82.5) | 62 (84.9) |  |
| ≥1 | 40 (17.5) | 11 (15.1) |  |
| CTP score, median (IQR) | 7.0 (6.0, 8.0) | 7.0 (6.0, 8.0) | 0.199 |
| MELD score, median (IQR) | 10.0 (8.0, 11.0) | 9.0 (8.0, 11.0) | 0.394 |
| WBC, median (IQR), 10⁹/L | 3.4 (2.3, 5.6) | 3.6 (2.5, 6.0) | 0.572 |
| Hemoglobin, median (IQR), g/L | 80.0 (69.0, 93.3) | 79.0 (61.0, 96.0) | 0.493 |
| Platelet, median (IQR), 10⁹/L | 69.5 (46.0, 95.3) | 78.0 (46.0, 99.0) | 0.572 |
| Neutrophil-to-lymphocyte ratio, median (IQR) | 3.3 (2.2, 5.5) | 2.9 (2.0, 4.7) | 0.196 |
| ALT, median (IQR), U/L | 19.0 (14.8, 32.0) | 19.0 (16.0, 31.0) | 0.963 |
| AST, median (IQR), U/L | 30.0 (22.0, 43.0) | 30.0 (22.0, 42.0) | 0.797 |
| Creatinine, median (IQR), umol/L | 54.3 (45.3, 70.7) | 51.1 (45.3, 69.0) | 0.781 |
| Serum sodium, median (IQR), mmol/L | 138.5 (136.6, 140.6) | 138.6 (136.5, 140.6) | 0.941 |
| BMI, median (IQR), kg/m2 | 22.5 (20.3, 24.8) | 22.5 (20.7, 24.6) | 0.892 |

IQR, interquartile range; CirCom, cirrhosis comorbidity; CTP, Child-Turcotte-Pugh; MELD, model for end-stage liver disease; WBC, white blood cell; ALT, alanine aminotransferase; AST, aspartate aminotransferase; BMI, body mass index.

**Supplementary Table 3.** Baseline characteristics before and after inverse probability of treatment weighting.

| **Variable** | **Before IPTW adjustment** | | | | | **After IPTW adjustment** | | | | |
| --- | --- | --- | --- | --- | --- | --- | --- | --- | --- | --- |
|  | **Overall** | **Non-LMM-VA** | **LMM-VA** | ***p* value** | **SMD** | **Overall** | **Non-LMM-VA** | **LMM-VA** | ***p* value** | **SMD** |
| n | 228 | 174 | 54 |  |  | 216.1 | 171.9 | 44.2 |  |  |
| Age, mean ± SD, years | 55.8 (11.1) | 54.6 (10.8) | 59.8 (10.9) | 0.002 | 0.484 | 56.1 (10.6) | 55.6 (10.7) | 58.0 (10.2) | 0.183 | 0.223 |
| Sex, n (%) |  |  |  |  |  |  |  |  |  |  |
| Male | 127 (55.7) | 100 (57.5) | 27 (50.0) | 0.419 | 0.150 | 113.5 (52.5) | 93.1 (54.2) | 20.4 (46.3) | 0.398 | 0.158 |
| Female | 101 (44.3) | 74 (42.5) | 27 (50.0) |  |  | 102.5 (47.5) | 78.8 (45.8) | 23.7 (53.7) |  |  |
| Etiology, n (%) |  |  |  |  |  |  |  |  |  |  |
| Viral hepatitis | 145 (63.6) | 119 (68.4) | 26 (48.1) | 0.018 | 0.475 | 134.3 (62.2) | 108.3 (63.0) | 26.1 (59.0) | 0.937 | 0.105 |
| Alcohol | 18 (7.9) | 11 (6.3) | 7 (13.0) |  |  | 17.1 (7.9) | 13.3 (7.8) | 3.8 (8.5) |  |  |
| Autoimmune | 29 (12.7) | 17 (9.8) | 12 (22.2) |  |  | 30.9 (14.3) | 23.4 (13.6) | 7.5 (17.1) |  |  |
| Others | 36 (15.8) | 27 (15.5) | 9 (16.7) |  |  | 33.7 (15.6) | 26.9 (15.6) | 6.8 (15.4) |  |  |
| Smoking, n (%) |  |  |  |  |  |  |  |  |  |  |
| No | 171 (75.0) | 134 (77.0) | 37 (68.5) | 0.280 | 0.192 | 162.6 (75.2) | 130.8 (76.1) | 31.7 (71.8) | 0.600 | 0.097 |
| Yes | 57 (25.0) | 40 (23.0) | 17 (31.5) |  |  | 53.5 (24.8) | 41.1 (23.9) | 12.4 (28.2) |  |  |
| Drinking, n (%) |  |  |  |  |  |  |  |  |  |  |
| No | 184 (80.7) | 143 (82.2) | 41 (75.9) | 0.412 | 0.154 | 175.3 (81.1) | 139.5 (81.1) | 35.8 (81.0) | 0.985 | 0.003 |
| Yes | 44 (19.3) | 31 (17.8) | 13 (24.1) |  |  | 40.8 (18.9) | 32.4 (18.9) | 8.4 (19.0) |  |  |
| Ascites, n (%) |  |  |  |  |  |  |  |  |  |  |
| No | 81 (35.5) | 69 (39.7) | 12 (22.2) | 0.030 | 0.384 | 76.4 (35.3) | 62.0 (36.1) | 14.4 (32.6) | 0.714 | 0.074 |
| Yes | 147 (64.5) | 105 (60.3) | 42 (77.8) |  |  | 139.7 (64.7) | 109.9 (63.9) | 29.8 (67.4) |  |  |
| Previous hepatic encephalopathy, n (%) |  |  |  |  |  |  |  |  |  |  |
| No | 220 (96.5) | 169 (97.1) | 51 (94.4) | 0.608 | 0.134 | 210.0 (97.2) | 167.1 (97.2) | 42.9 (97.1) | 0.973 | 0.004 |
| Yes | 8 (3.5) | 5 (2.9) | 3 (5.6) |  |  | 6.1 (2.8) | 4.8 (2.8) | 1.3 (2.9) |  |  |
| Portal vein thrombosis, n (%) |  |  |  |  |  |  |  |  |  |  |
| No | 145 (63.6) | 111 (63.8) | 34 (63.0) | 1.000 | 0.017 | 139.1 (64.4) | 110.7 (64.4) | 28.3 (64.2) | 0.979 | 0.005 |
| Yes | 83 (36.4) | 63 (36.2) | 20 (37.0) |  |  | 77.0 (35.6) | 61.2 (35.6) | 15.8 (35.8) |  |  |
| CirCom score, n (%) |  |  |  |  |  |  |  |  |  |  |
| 0 | 188 (82.5) | 139 (79.9) | 49 (90.7) | 0.104 | 0.310 | 179.5 (83.1) | 141.6 (82.4) | 37.9 (85.7) | 0.672 | 0.091 |
| ≥1 | 40 (17.5) | 35 (20.1) | 5 (9.3) |  |  | 36.6 (16.9) | 30.3 (17.6) | 6.3 (14.3) |  |  |
| Previous endoscopic or NSBB treatment, n (%) |  |  |  |  |  |  |  |  |  |  |
| No | 120 (52.6) | 100 (57.5) | 20 (37.0) | 0.013 | 0.418 | 111.0 (51.4) | 90.1 (52.4) | 20.9 (47.2) | 0.580 | 0.104 |
| Yes | 108 (47.4) | 74 (42.5) | 34 (63.0) |  |  | 105.1 (48.6) | 81.8 (47.6) | 23.3 (52.8) |  |  |
| CTP score, median (IQR) | 7.0 (6.0, 8.0) | 7.0 (6.0, 8.0) | 7.5 (6.3, 8.0) | 0.011 | 0.392 | 7.0 (6.0, 8.0) | 7.0 (6.0, 8.0) | 7.0 (6.0, 8.0) | 0.796 | 0.060 |
| CTP classification, n (%) |  |  |  |  |  |  |  |  |  |  |
| A | 80 (35.1) | 66 (37.9) | 14 (25.9) | 0.125 | 0.310 | 78.1 (36.1) | 60.3 (35.1) | 17.8 (40.4) | 0.656 | 0.171 |
| B | 136 (59.6) | 101 (58.0) | 35 (64.8) |  |  | 124.4 (57.6) | 101.8 (59.2) | 22.6 (51.2) |  |  |
| C | 12 (5.3) | 7 (4.0) | 5 (9.3) |  |  | 13.6 (6.3) | 9.9 (5.7) | 3.7 (8.4) |  |  |
| MELD score, median (IQR) | 10.0 (8.0, 11.0) | 10.0 (8.0, 11.0) | 9.5 (8.0, 11.0) | 0.830 | 0.080 | 10.0 (8.0, 11.0) | 10.0 (8.0, 11.0) | 10.0 (8.0, 11.2) | 0.475 | 0.173 |
| WBC, median (IQR), 10⁹/L | 3.4 (2.3, 5.6) | 3.2 (2.4, 4.9) | 3.8 (2.1, 6.5) | 0.458 | 0.151 | 3.5 (2.4, 6.0) | 3.5 (2.5, 5.2) | 3.9 (2.1, 6.8) | 0.529 | 0.166 |
| Hemoglobin, median (IQR), g/L | 80.0 (69.0, 93.3) | 80.0 (72.0, 94.0) | 81.0 (65.3, 91.8) | 0.659 | 0.105 | 80.0 (68.4, 92.9) | 80.0 (70.9, 93.1) | 79.8 (64.8, 90.8) | 0.746 | 0.116 |
| Platelet, median (IQR), 10⁹/L | 69.5 (46.0, 95.3) | 71.0 (46.3, 95.8) | 66.5 (46.3, 93.8) | 0.871 | 0.065 | 71.0 (46.0, 94.9) | 72.2 (47.0, 95.7) | 66.0 (45.0, 92.2) | 0.653 | 0.042 |
| Neutrophil-to-lymphocyte ratio, median (IQR) | 3.3 (2.2, 5.5) | 3.1 (2.1, 5.0) | 4.1 (3.1, 7.6) | 0.002 | 0.343 | 3.3 (2.1, 5.5) | 3.3 (2.1, 5.1) | 3.6 (2.1, 7.3) | 0.448 | 0.099 |
| INR, median (IQR) | 1.2 (1.1, 1.3) | 1.2 (1.1, 1.3) | 1.2 (1.2, 1.4) | 0.290 | 0.132 | 1.2 (1.1, 1.3) | 1.2 (1.1, 1.3) | 1.2 (1.2, 1.4) | 0.459 | 0.114 |
| TBIL, median (IQR), μmol/L | 24.3 (16.4, 32.4) | 24.1 (16.6, 31.7) | 25.2 (14.3, 35.0) | 0.782 | 0.285 | 24.1 (16.1, 32.8) | 24.7 (16.5, 32.7) | 18.6 (14.0, 33.3) | 0.386 | 0.087 |
| ALT, median (IQR), U/L | 19.0 (14.8, 32.0) | 19.0 (14.0, 31.0) | 20.5 (15.0, 37.8) | 0.410 | 0.132 | 19.0 (14.0, 31.8) | 19.0 (14.0, 31.0) | 20.1 (15.0, 32.6) | 0.438 | 0.017 |
| AST, median (IQR), U/L | 30.0 (22.0, 43.0) | 30.0 (23.0, 41.0) | 31.5 (22.0, 51.3) | 0.203 | 0.168 | 30.0 (22.0, 42.7) | 30.0 (22.0, 41.0) | 30.0 (22.0, 45.1) | 0.518 | 0.051 |
| Albumin, mean ± SD, g/L | 32.7 (5.4) | 32.9 (5.4) | 32.0 (5.2) | 0.311 | 0.160 | 32.7 (5.3) | 32.7 (5.4) | 32.7 (4.9) | 1.000 | <0.001 |
| Creatinine, median (IQR), μmol/L | 54.3 (45.3, 70.7) | 54.2 (45.4, 67.7) | 56.6 (43.5, 79.8) | 0.378 | 0.193 | 54.2 (44.4, 70.1) | 54.2 (45.3, 67.3) | 55.4 (41.2, 80.2) | 0.609 | 0.196 |
| Serum sodium, mean ± SD, mmol/L | 138.5 (3.0) | 138.8 (2.9) | 137.9 (3.2) | 0.054 | 0.295 | 138.6 (3.0) | 138.6 (3.0) | 138.4 (3.0) | 0.759 | 0.055 |
| Blood ammonia, median (IQR), μmol/L | 59.5 (46.0, 80.0) | 61.5 (47.3, 80.8) | 57.0 (43.3, 73.0) | 0.187 | 0.244 | 59.0 (45.0, 79.0) | 59.8 (46.3, 80.0) | 57.0 (43.0, 73.0) | 0.331 | 0.214 |
| BMI, median (IQR), kg/m^2^ | 22.5 (20.3, 24.8) | 22.9 (20.9, 25.6) | 20.4 (19.0, 23.1) | <0.001 | 0.648 | 22.5 (20.3, 25.0) | 22.9 (20.8, 25.6) | 20.3 (18.9, 23.2) | <0.001 | 0.658 |
| SMI, median (IQR), cm^2^/m^2^ | 41.6 (35.7, 48.5) | 44.7 (38.4, 50.9) | 31.3 (28.7, 37.6) | <0.001 | 1.539 | 41.4 (35.5, 48.5) | 43.9 (37.4, 50.7) | 31.0 (28.4, 37.6) | <0.001 | 1.486 |
| VATI, median (IQR), cm^2^/m^2^ | 28.1 (18.1, 40.5) | 28.7 (17.7, 40.9) | 25.3 (19.2, 38.8) | 0.964 | 0.033 | 28.4 (19.3, 42.2) | 29.0 (18.5, 42.4) | 25.3 (21.3, 39.5) | 0.956 | 0.039 |
| SATI, median (IQR), cm^2^/m^2^ | 32.5 (19.4, 49.1) | 37.1 (23.1, 51.3) | 22.7 (12.1, 34.3) | <0.001 | 0.626 | 34.0 (21.6, 49.4) | 37.6 (23.7, 50.8) | 29.4 (15.4, 34.3) | <0.001 | 0.593 |
| VSR, median (IQR) | 0.9 (0.6, 1.3) | 0.9 (0.6, 1.1) | 1.4 (0.9, 1.9) | <0.001 | 0.611 | 0.9 (0.6, 1.2) | 0.9 (0.6, 1.1) | 1.2 (0.8, 1.7) | <0.001 | 0.391 |
| Shunt position, n (%) |  |  |  |  |  |  |  |  |  |  |
| Left portal vein | 174 (76.3) | 135 (77.6) | 39 (72.2) | 0.262 | 0.236 | 165.0 (76.4) | 133.8 (77.8) | 31.2 (70.7) | 0.519 | 0.197 |
| Right portal vein | 33 (14.5) | 26 (14.9) | 7 (13.0) |  |  | 33.0 (15.3) | 25.7 (15.0) | 7.3 (16.6) |  |  |
| Portal vein bifurcation | 21 (9.2) | 13 (7.5) | 8 (14.8) |  |  | 18.0 (8.3) | 12.4 (7.2) | 5.6 (12.7) |  |  |
| Collateral embolization, n (%) |  |  |  |  |  |  |  |  |  |  |
| No | 22 (9.6) | 17 (9.8) | 5 (9.3) | 1.000 | 0.017 | 19.5 (9.0) | 16.4 (9.5) | 3.1 (7.0) | 0.584 | 0.091 |
| Yes | 206 (90.4) | 157 (90.2) | 49 (90.7) |  |  | 196.6 (91.0) | 155.5 (90.5) | 41.1 (93.0) |  |  |
| PPG (before TIPS stent), median (IQR), mmHg | 35.0 (31.0, 40.0) | 34.0 (31.0, 39.8) | 35.0 (30.3, 42.8) | 0.343 | 0.176 | 34.0 (30.0, 40.0) | 35.0 (31.0, 40.0) | 34.0 (29.0, 40.0) | 0.523 | 0.114 |
| PPG (after TIPS stent), median (IQR), mmHg | 12.0 (9.0, 15.0) | 12.0 (9.0, 15.0) | 12.0 (9.3, 15.0) | 0.445 | 0.104 | 12.0 (9.0, 15.0) | 12.0 (9.0, 15.0) | 10.1 (8.0, 14.0) | 0.395 | 0.151 |
| PPG reduction, mean ± SD, mmHg | 23.2 (5.5) | 23.0 (5.4) | 23.8 (5.9) | 0.386 | 0.131 | 23.0 (5.5) | 23.0 (5.2) | 22.9 (6.4) | 0.901 | 0.025 |

Between-group comparisons were performed using the t-test or Mann–Whitney U test for continuous variables, and the χ² test or Fisher’s exact test for categorical variables, as appropriate.

LMM-VA, low muscle mass and visceral adiposity; IPTW, inverse probability of treatment weighting; TIPS, transjugular intrahepatic portosystemic shunt; SD, standard deviation; SMD, standardized mean difference; CirCom, cirrhosis comorbidity; NSBB, non-selective beta-blockers; CTP, Child-Turcotte-Pugh; IQR, interquartile range; MELD, model for end-stage liver disease; WBC, white blood cell; INR, international normalized ratio; TBIL, total bilirubin; ALT, alanine aminotransferase; AST, aspartate aminotransferase; BMI, body mass index; SMI, skeletal muscle index; VATI, visceral adipose tissue index; SATI, subcutaneous adipose tissue index; VSR, visceral to subcutaneous ratio; PPG, portal pressure gradient.

**Supplementary Table 4. Sensitivity analyses of the association between LMM-VA and all-cause mortality based on multivariable adjusted models.**

| **Variable** | **Events per variable** | **Sensitivity analysis 1^a^** | | **Sensitivity analysis 2^b^** | | **Sensitivity analysis 3^c^** | | **Sensitivity analysis 4^d^** | | **Sensitivity analysis 5^e^** | | |
| --- | --- | --- | --- | --- | --- | --- | --- | --- | --- | --- | --- | --- |
|  |  | **HR (95% CI)** | ***p* value** | **HR (95% CI)** | ***p* value** | **HR (95% CI)** | ***p* value** | **HR (95% CI)** | ***p* value** | **HR (95% CI)** | ***p* value** |  |
| LMM-VA | | | |  |  |  |  |  |  |  |  |  |
| No | 14 (174) | 1.00 (reference) | | 1.00 (reference) | | 1.00 (reference) | | 1.00 (reference) | | 1.00 (reference) | | |
| Yes | 12 (54) | 3.21 (1.33-7.68) | 0.010 | 3.03 (1.26-7.30) | 0.014 | 3.51 (1.20-10.23) | 0.021 | 3.40 (1.25-9.67) | 0.012 | 3.94 (1.58-9.86) | 0.003 |  |
| Age, years | 26 (228) | 1.26 (0.85-1.91) | 0.247 | 1.02 (0.98-1.06) | 0.277 | 1.02 (0.97-1.07) | 0.490 | 1.34 (0.82-2.26) | 0.242 | 1.51 (1.01-2.26) | 0.045 |  |
| Sex |  |  |  |  |  |  |  |  |  |  |  |  |
| Male | 12 (127) | - | | - | | 1.00 (reference) | | - | | - | |  |
| Female | 14 (101) | - | | - | | 1.67 (0.69-4.06) | 0.256 | - | | - | |  |
| Etiology |  |  |  |  |  |  |  |  |  |  |  |  |
| Viral hepatitis | 17 (145) | - | | - | | 1.00 (reference) | | - | | - | |  |
| Alcohol | 2 (18) | - | | - | | 1.66 (0.28-9.78) | 0.575 | - | | - | |  |
| Autoimmune | 2 (29) | - | | - | | 0.38 (0.07-2.07) | 0.262 | - | | - | |  |
| Others | 5 (36) | - | | - | | 0.83 (0.25-2.82) | 0.769 | - | | - | |  |
| CirCom score | |  |  |  |  |  |  |  |  |  |  |  |
| 0 | 20 (188) | 1.00 (reference) | | 1.00 (reference) | | 1.00 (reference) | | 1.00 (reference) | | 1.00 (reference) | | |
| ≥1 | 6 (40) | 2.16 (0.78-5.34) | 0.130 | 2.24 (0.83-6.01) | 0.110 | 1.95 (0.69-5.52) | 0.207 | 1.92 (0.52-6.60) | 0.224 | 2.04 (0.77-5.39) | 0.152 |  |
| MELD score | 26 (228) | 1.17 (0.77-1.73) | 0.464 | 1.04 (0.90-1.19) | 0.627 | 1.05 (0.89-1.24) | 0.585 | 1.14 (0.69-1.77) | 0.560 | 1.16 (0.78-1.73) | 0.452 |  |
| Hemoglobin, g/L | 26 (228) | 0.72 (0.45-1.10) | 0.136 | 0.98 (0.97-1.00) | 0.131 | - | | 0.67 (0.35-1.16) | 0.172 | 0.67 (0.42-1.06) | 0.085 |  |
| Serum sodium, mmol/L | 26 (228) | 0.79 (0.53-1.13) | 0.200 | 0.91 (0.80-1.04) | 0.164 | - | | 0.76 (0.48-1.19) | 0.186 | 0.83 (0.55-1.24) | 0.367 |  |
| Blood ammonia, umol/L | 26 (228) | - | | - | | 1.01 (0.99-1.03) | 0.259 | - | | - | |  |
| PPG (before TIPS stent), mmHg | 26 (228) | 0.73 (0.38-1.31) | 0.301 | 0.95 (0.87-1.05) | 0.344 | - | | 0.65 (0.31-1.16) | 0.156 | 0.72 (0.38-1.35) | 0.308 |  |
| PPG reduction, mmHg | 26 (228) | 0.84 (0.44-1.62) | 0.590 | 0.97 (0.86-1.09) | 0.600 | - | | 0.90 (0.37-2.26) | 0.800 | 0.88 (0.46-1.70) | 0.711 |  |

^a^ Multivariable model based on Firth correction regression;

^b^ Multivariable Cox regression model with time-zero redefined as the date of TIPS placement;

^c^ Multivariable Cox regression model with Inverse Probability of Treatment Weighting. Variables included in the multivariable model were selected based on weighted SMD > 0.2 and clinically recognized strong prognostic factors;

^d^ Multivariable Cox regression model via bootstrapping with 1000 iterations;

^e^ Low muscle mass and visceral adiposity were defined using data-driven sex-specific cut-off values based on the maximum Youden index.

LMM-VA, low muscle mass and visceral adiposity; HR, hazard ratio; CI, confidence interval; MELD, model for end-stage liver disease; CirCom, cirrhosis comorbidity; PPG, portal pressure gradient; TIPS, transjugular intrahepatic portosystemic shunt.

**Supplementary Table 5.** Univariable and multivariable Cox regression analyses of the association between low muscle mass and visceral adiposity and all-cause mortality stratified by the presence of low muscle mass and visceral adiposity.

| **Variable** | **Univariable analysis** | | **Multivariable analysis** | |
| --- | --- | --- | --- | --- |
|  | **HR (95% CI)** | ***p* value** | **HR (95% CI)** | ***p* value** |
| Age, years | 1.56 (1.02-2.38) | 0.041 | 1.21 (0.80-1.83) | 0.362 |
| Sex |  |  |  |  |
| Male | 1.00 (reference) | |  |  |
| Female | 1.33 (0.61-2.88) | 0.470 |  |  |
| Etiology |  |  |  |  |
| Viral hepatitis | 1.00 (reference) | |  |  |
| Alcohol | 1.09 (0.25-4.75) | 0.904 |  |  |
| Autoimmune | 0.60 (0.14-2.61) | 0.497 |  |  |
| Others | 1.05 (0.39-2.85) | 0.927 |  |  |
| Smoking | 1.06 (0.42-2.64) | 0.908 |  |  |
| Drinking | 1.89 (0.79-4.53) | 0.152 |  |  |
| Ascites | 1.52 (0.66-3.50) | 0.329 |  |  |
| Previous hepatic encephalopathy | NC^a^ | 0.997 |  |  |
| Portal vein thrombosis | 0.88 (0.39-1.97) | 0.751 |  |  |
| CirCom score |  |  |  |  |
| 0 | 1.00 (reference) | | 1.00 (reference) | |
| ≥1 | 1.30 (0.52-3.23) | 0.579 | 2.41 (0.87-6.68) | 0.090 |
| Previous endoscopic or NSBB treatment | 1.00 (0.46-2.16) | 0.999 |  |  |
| CTP score | 1.22 (0.87-1.73) | 0.249 |  |  |
| CTP classification |  |  |  |  |
| A | 1.00 (reference) |  |  |  |
| B | 1.93 (0.80-4.66) | 0.143 |  |  |
| C | 1.13 (0.14-9.23) | 0.906 |  |  |
| MELD score | 1.12 (0.75-1.66) | 0.576 | 1.12 (0.73-1.72) | 0.592 |
| Muscle and adipose tissue abnormalities |  |  |  |  |
| Normal | 1.00 (reference) | | 1.00 (reference) | |
| Isolated visceral adiposity | 0.65 (0.08-5.59) | 0.698 | 0.56 (0.06-5.12) | 0.609 |
| Isolated low muscle mass | 1.36 (0.45-4.18) | 0.587 | 1.63 (0.50-5.30) | 0.420 |
| Low muscle mass and visceral adiposity | 4.12 (1.44-11.76) | 0.008 | 4.14 (1.28-13.38) | 0.018 |
| WBC, 10⁹/L | 1.12 (0.79-1.59) | 0.516 |  |  |
| Hemoglobin, g/L | 0.62 (0.40-0.97) | 0.037 | 0.70 (0.45-1.11) | 0.135 |
| PLT, 10⁹/L | 1.10 (0.83-1.47) | 0.505 |  |  |
| Neutrophil-to-lymphocyte ratio | 1.25 (0.89-1.76) | 0.202 |  |  |
| INR | 1.03 (0.70-1.51) | 0.874 |  |  |
| TBIL, umol/L | 1.04 (0.75-1.46) | 0.801 |  |  |
| ALT, U/L | 0.98 (0.70-1.36) | 0.894 |  |  |
| AST, U/L | 1.15 (0.87-1.36) | 0.321 |  |  |
| Albumin, g/L | 0.80 (0.54-1.19) | 0.275 |  |  |
| Creatinine, umol/L | 1.35 (0.95-1.92) | 0.096^b^ |  |  |
| Serum sodium, mmol/L | 0.71 (0.50-1.02) | 0.063 | 0.75 (0.51-1.11) | 0.156 |
| Blood ammonia, umol/L | 1.29 (0.92-1.81) | 0.141 |  |  |
| BMI, kg/m^2^ | 0.75 (0.49-1.15) | 0.185 |  |  |
| SMI, cm²/m² | 0.67 (0.47-0.95) | 0.023^c^ |  |  |
| VATI, cm²/m² | 0.70 (0.43-1.12) | 0.137 |  |  |
| SATI, cm²/m² | 0.74 (0.47-1.17) | 0.203 |  |  |
| VSR | 1.04 (0.69-1.58) | 0.850 |  |  |
| Shunt position, n (%) |  |  |  |  |
| Left portal vein | 1.00 (reference) | |  |  |
| Right portal vein | 0.63 (0.15-2.71) | 0.538 |  |  |
| Portal vein bifurcation | 2.04 (0.70-5.99) | 0.193 |  |  |
| Collateral embolization | 0.46 (0.17-1.21) | 0.114 |  |  |
| PPG (before TIPS stent), mmHg | 0.67 (0.44-1.00) | 0.052 | 0.70 (0.37-1.31) | 0.265 |
| PPG (after TIPS stent), mmHg | 0.89 (0.60-1.31) | 0.558 |  |  |
| PPG reduction, mmHg | 0.67 (0.44-1.02) | 0.064 | 0.84 (0.44-1.63) | 0.614 |

TIPS, transjugular intrahepatic portosystemic shunt; HR, hazard ratio; CI, confidence interval; NC, not calculable; CirCom, cirrhosis comorbidity; NSBB, non-selective beta-blockers‌; CTP, Child-Turcotte-Pugh; MELD, model for end-stage liver disease; WBC, white blood cell; INR, international normalized ratio; TBIL, total bilirubin; ALT, alanine aminotransferase; AST, aspartate aminotransferase; BMI, body mass index; SMI, skeletal muscle index; VATI, visceral adipose tissue index; SATI, subcutaneous adipose tissue index; VSR, the visceral to subcutaneous ratio; PPG, portal pressure gradient.

^a^Hazard ratio and confidence interval not estimable due to sparse data.

^b^Creatinine was not included in the multivariate analysis because it is a component of the MELD score.

^c^SMI was not included in the multivariate analysis because it was a component of the definition of low muscle mass and visceral adiposity.

**Supplementary Table 6. Model performance of LMM-VA combined with liver function models in predicting all-cause mortality among patients with cirrhosis undergoing TIPS.**

| **Model** | **Discrimination** | **Calibration** | **Model improvement** | |
| --- | --- | --- | --- | --- |
|  | **C-index (95% CI)** | **Brier score** | **NRI (95CI)** | ***p* value** |
| MELD score | 0.53 (0.41-0.66) | 0.093 | reference |  |
| MELD score+LMM-VA | 0.66 (0.55-0.78) | 0.088 | 0.05 (0.01-0.14) | 0.008 |
| CTP score | 0.63 (0.53-0.73) | 0.093 | reference |  |
| CTP score+LMM-VA | 0.69 (0.59-0.80) | 0.088 | 0.05 (0.01-0.13) | 0.010 |

LMM-VA, low muscle mass and visceral adiposity; TIPS, transjugular intrahepatic portosystemic shunt; CI, confidence interval; NRI, net reclassification improvement; MELD, model for end-stage liver disease; CTP, Child-Turcotte-Pugh.

**Supplementary Table 7.** Univariable and multivariable competing risk analysis for predictors of liver-related death in patients with cirrhosis undergoing TIPS.

| **Variable** | **Univariable analysis** | | **Multivariable analysis** | |
| --- | --- | --- | --- | --- |
|  | **sHR (95% CI)** | ***p* value** | **sHR (95% CI)** | ***p* value** |
| Age, years | 1.53 (0.87-2.66) | 0.137 |  |  |
| Sex |  |  |  |  |
| Male | 1.00 (reference) | |  |  |
| Female | 0.90 (0.37-2.21) | 0.820 |  |  |
| Etiology |  |  |  |  |
| Viral hepatitis | 1.00 (reference) | |  |  |
| Alcohol | 1.57 (0.36-6.82) | 0.547 |  |  |
| Autoimmune | 0.38 (0.05-2.77) | 0.340 |  |  |
| Others | 1.58 (0.56-4.44) | 0.386 |  |  |
| Smoking | 1.55 (0.59-4.06) | 0.374 |  |  |
| Drinking | 2.21 (0.84-5.83) | 0.111 |  |  |
| Ascites | 2.81 (1.01-7.81) | 0.048^a^ |  |  |
| Portal vein thrombosis | 0.88 (0.36-2.17) | 0.785 |  |  |
| CirCom score |  |  |  |  |
| 0 | 1.00 (reference) | | 1.00 (reference) | |
| ≥1 | 1.41 (0.52-3.79) | 0.502 | 1.68 (0.60-4.71) | 0.320 |
| Previous endoscopic or NSBB treatment | 0.61 (0.24-1.53) | 0.291 |  |  |
| CTP score | 1.30 (0.95-1.77) | 0.106 |  |  |
| CTP classification |  |  |  |  |
| A | 1.00 (reference) | | 1.00 (reference) | |
| B | 2.61 (1.04-6.56) | 0.041 | 2.08 (0.78-5.53) | 0.140 |
| C | 0.99 (0.12-8.26) | 0.989 | 0.80 (0.08-8.20) | 0.850 |
| MELD score | 1.03 (0.66-1.60) | 0.901 |  |  |
| Low muscle mass and visceral adiposity | 4.25 (1.80-10.03) | 0.001 | 4.18 (1.69-10.30) | 0.002 |
| WBC, 10⁹/L | 1.04 (0.59-1.86) | 0.884 |  |  |
| Hemoglobin, g/L | 0.50 (0.27-0.92) | 0.025 | 0.54 (0.28-1.07) | 0.077 |
| Platelet, 10⁹/L | 0.89 (0.56-1.40) | 0.616 |  |  |
| Neutrophil-to-lymphocyte ratio | 1.36 (0.93-1.99) | 0.109 |  |  |
| INR | 1.12 (0.76-1.65) | 0.569 |  |  |
| TBIL, umol/L | 0.92 (0.61-1.41) | 0.707 |  |  |
| ALT, U/L | 0.89 (0.60-1.31) | 0.554 |  |  |
| AST, U/L | 1.05 (0.76-1.43) | 0.785 |  |  |
| Albumin, g/L | 0.82 (0.52-1.28) | 0.381 |  |  |
| Creatinine, umol/L | 1.48 (1.06-2.05) | 0.021 | 1.35 (1.00-1.83) | 0.051 |
| Serum sodium, mmol/L | 0.73 (0.48-1.10) | 0.129 |  |  |
| Blood ammonia, umol/L | 1.27 (0.85-1.89) | 0.246 |  |  |
| BMI, kg/m^2^ | 0.82 (0.50-1.33) | 0.418 |  |  |
| SMI, cm²/m² | 0.71 (0.47-1.07) | 0.102 |  |  |
| VATI, cm²/m² | 0.84 (0.54-1.33) | 0.461 |  |  |
| SATI, cm²/m² | 0.75 (0.46-1.21) | 0.233 |  |  |
| VSR | 1.12 (0.89-1.42) | 0.347 |  |  |
| Shunt position, n (%) |  |  |  |  |
| Left portal vein | 1.00 (reference) | |  |  |
| Right portal vein | 0.36 (0.05-2.73) | 0.320 |  |  |
| Portal vein bifurcation | 2.04 (0.61-6.82) | 0.248 |  |  |
| Collateral embolization | 0.64 (0.19-2.14) | 0.472 |  |  |
| PPG (before TIPS stent), mmHg | 0.69 (0.49-0.99) | 0.041 | 0.83 (0.47-1.47) | 0.520 |
| PPG (after TIPS stent), mmHg | 0.96 (0.68-1.35) | 0.814 |  |  |
| PPG reduction, mmHg | 0.66 (0.43-1.02) | 0.062 | 0.75 (0.37-1.53) | 0.430 |

TIPS, transjugular intrahepatic portosystemic shunt; sHR, subdistribution hazard ratio; CI, confidence interval; CirCom, cirrhosis comorbidity; NSBB, non-selective beta-blockers‌; CTP, Child-Turcotte-Pugh; MELD, model for end-stage liver disease; WBC, white blood cell; INR, international normalized ratio; TBIL, total bilirubin; ALT, alanine aminotransferase; AST, aspartate aminotransferase; BMI, body mass index; SMI, skeletal muscle index; VATI, visceral adipose tissue index; SATI, subcutaneous adipose tissue index; VSR, the visceral to subcutaneous ratio; PPG, portal pressure gradient.

^a^Ascites was not included in the multivariate analysis because it is a component of the CTP classification.
